# Screen-Printed Stretchable Supercapacitors Based on Tin Sulfide-Decorated Face-Mask-Derived Activated Carbon Electrodes with High Areal Energy Density

**DOI:** 10.1021/acsaem.3c02902

**Published:** 2024-04-18

**Authors:** Kiran Kumar Reddy Reddygunta, Lidija Šiller, Aruna Ivaturi

**Affiliations:** † Smart Materials Research and Device Technology (SMaRDT) Group, Department of Pure and Applied Chemistry, University of Strathclyde, Thomas Graham Building, Glasgow G1 1XL, U.K.; ‡ School of Engineering, Newcastle University, Newcastle upon Tyne NE1 7RU, U.K.

**Keywords:** activated carbon, tin sulfide, solid-state gel electrolyte, stretchable supercapacitor, screen printing

## Abstract

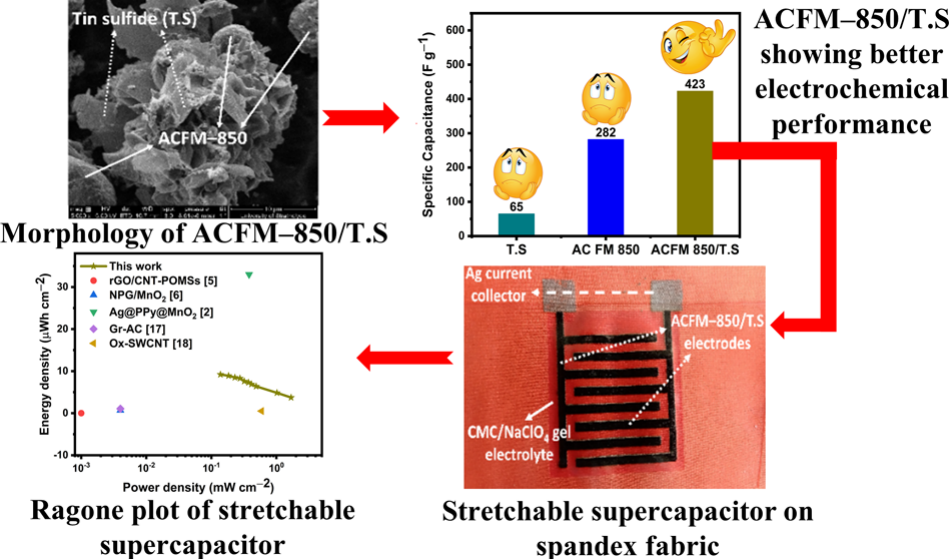

In this work, tin sulfide nanosheets decorated on face-mask-derived activated carbon have been explored as electrode material for electrochemical supercapacitors. A hydrothermal route was employed to grow tin sulfide on the surface and inside of high-surface-area face-mask-derived activated carbon, activated at 850 °C, to produce a hierarchical interconnected porous composite (ACFM-850/TS) structure. The presence of tin sulfide in the porous carbon framework exposed the surface active sites for rapid adsorption/desorption of electrolyte ions and ensured high utilization of the porous carbon surface. Furthermore, the porous ACFM-850 framework prevented the stacking/agglomeration of tin sulfide sheets, thereby enhancing the charge-transport kinetics in the composite electrodes. Benefiting from the synergistic effect of tin sulfide and ACFM-850, the resulting ACFM-850/TS composite exhibited an attractive specific capacitance of 423 F g^–1^ at a 0.5 A g^–1^ current density and superior rate capability (71.3% at a 30 A g^–1^ current density) in a 1.0 M Na_2_SO_4_ electrolyte. In addition, we fabricated a planar symmetric interdigitated supercapacitor on a stretchable Spandex fabric using an ACFM-850/TS composite electrode and carboxymethyl cellulose/NaClO_4_ as a solid-state gel electrolyte employing a scalable screen-printing process. The as-prepared stretchable supercapacitors displayed an ultrahigh energy density of 9.2 μWh cm^–2^ at a power density of 0.13 mW cm^–2^. In addition, they exhibited an excellent cyclic stability of 64% even after 10,000 charge–discharge cycles and 42% after 1000 continuous stretch (at 25% stretching)/release cycles. Such screen-printed interdigitated planar supercapacitors with activated carbon composite electrodes and a solid-state gel electrolyte act as promising low-cost energy-storage devices for wearable and flexible integrated electronic devices.

## 1 Introduction

Supercapacitors offer a higher energy density than regular capacitors and a higher power density than standard lithium batteries. Many research groups have focused on the manufacture of low-cost, flexible, and stretchable supercapacitors, particularly for use in wearable technology and the Internet of Things (IoT).^1,2^ Flexible and stretchable supercapacitors can be constructed either in sandwich or planar (interdigitated/electrode finger arrays) configurations.^3,4^ Planar (interdigitated) electrodes and devices are mainly fabricated using printed electronic techniques such as photolithography, plasma etching, and direct laser writing techniques. For example, Zhu et al.^5^ prepared a reduced graphene oxide and carbon nanotube (rGO/CNT) electrode-based planar microsupercapacitor using the photolithography technique on a silicon substrate. The areal capacitance of the rGO/CNT-based interdigitated supercapacitor was found to be 28.62 mF cm^–2^ with an energy density of 0.004 μWh cm^–2^ at a power density of 0.001 mW cm^–2^.^5^ Shi et al.^6^ fabricated a planar microsupercapacitor based on nanoporous gold/manganese oxide (NPG/MnO_2_) hybrid electrodes via the plasma etching technique. The as-prepared supercapacitor displayed a relatively high areal energy density of 0.69 μWh cm^–2^ at a 0.004 mW cm^–2^ power density in a poly(vinyl alcohol) (PVA)/LiCl solid-state electrolyte.^6^ Song et al.^7^ employed a laser writing process to fabricate a laser-scribed interdigitated supercapacitor that delivered an areal capacitance of 0.72 mF cm^–2^ at a current density of 0.075 mA cm^–2^ in a potassium polyacrylate–potassium hydroxide (PAAK/KOH) gel electrolyte.^7^ However, techniques like direct laser writing, photolithography, and plasma etching processes are inconvenient for producing supercapacitors on a large scale due to their time-consuming process, high cost, and substrate limitations. Screen printing, as an emerging printed electronic fabrication technology, has significant potential to accomplish the objective of low-cost, large-scale manufacturing of flexible electronics.^8^

Screen printing is a surface patterning process that involves depositing inks or gels onto a substrate through microscopic pores in a mesh screen. It has significant promise for large-scale production of interdigitated supercapacitors due to its low cost, compatibility for a range of substrates, diversity of patterned screens, and simple operation.^8,9^ By formulating inks containing functional materials, screen printing has been shown to have great potential for a variety of flexible electronic applications such as sensors,^10^ touch screen displays,^11^ solar cells,^12^ light-emitting diodes (LEDs),^13^ radio frequency identification (RFID) tags,^14^ electronic textiles,^15^ batteries,^16^ and supercapacitors.^1^ Additionally, screen printing provides precise control over the electrode thickness and geometry and can be used to print on a variety of flexible substrates, such as paper, textiles, and plastics.^9^ To date, the bulk of screen-printed supercapacitors has been fabricated on various substrates using graphene,^17^ carbon nanotubes (CNTs),^18^ and conductive polymer^19^-based functional electrode inks. Chen et al.^17^ fabricated graphene/activated carbon-based screen-printed microsupercapacitors with an areal capacitance of 12.5 mF cm^–2^ in a PVA/H_2_SO_4_ electrolyte. A screen-printed carbon nanotube-based planar supercapacitor has been fabricated on A4 paper by Jo et al.^18^ The paper-based flexible supercapacitor exhibited a high energy density of 0.51 μWh cm^–2^ at a power density of 0.59 mW cm^–2^ and retained 85% of its capacitance after 10,000 cycles. Liu and coworkers fabricated an all-printed stretchable asymmetric supercapacitor consisting of a silver/polypyrrole/manganese dioxide (Ag@PPy@MnO_2_) cathode and an activated carbon anode on a stretchable textile, which exhibited an excellent energy density of 33 μWh cm^–2^ at a 0.38 mW cm^–2^ power density.^2^ Although screen-printed graphene or carbon-nanotube-based supercapacitors demonstrate excellent electrochemical performance, these materials are typically derived from fossil fuels and involve complex fabrication processes and chemical treatments, increasing manufacturing costs for a large area and mass production of supercapacitors. On the other hand, conducting polymer-based electrode materials are inexpensive and possess high pseudocapacitance and excellent flexibility but their low conductivity and poor cycling stability limits their practical application.^20,21^ As a result, the development of affordable and highly conductive functional electrodes for planar supercapacitors is still a pressing issue.

Transition-metal sulfides such as molybdenum sulfide, tin sulfide (TS), nickel sulfide, etc., have been identified as promising electrodes for supercapacitors due to their exceptional theoretical capacitance, superior energy density, exceptional chemical stability, and increased electrochemical activity.^22−25^ Tin sulfide, in particular, is considered the most important semiconductor of the transition-metal sulfide family with high theoretical specific capacitance and high charge carrier mobility.^26^ However, these materials suffer from significant volume expansion and restacking during the continuous charge–discharge process, which reduces the conductivity, charge-transport kinetics, and cyclic stability.^24,27^ Several strategies, such as combining tin sulfide with graphene, CNTs, and g-C_3_N_4_ materials, were employed to enhance the electrochemical performance and prevent the restacking of tin sulfide materials.^24,28,29^ Although, tin sulfide showed excellent performance when combined with graphene/CNTs, the production of graphene/CNTs in large quantities is still a challenging issue and requires fossil fuels. Therefore, combining tin sulfide with other carbon-based materials, such as activated carbon, is considered a promising alternative to overcome the obstacles posed by graphene/CNTs.

Plastic or biomass waste-derived activated carbons are considered attractive material for supercapacitor electrodes due to their superior electrical conductivity, high specific surface area (SSA), and widespread availability.^30,31^ The widespread usage of plastic-based face masks during the COVID-19 outbreak has resulted in harmful plastic waste.^32^ The face masks are made from single-use polypropylene (PP) fabrics, which cannot be recycled, and the majority of them are incinerated or disposed of as regular plastic waste, which is neither environment-friendly nor sustainable.^33,34^ Hence, upcycling these waste face masks into activated carbons is a promising approach for producing high-value-added carbon electrode materials for supercapacitor applications. Yang et al.^33^ prepared carbon nanotubes (CNTs) from waste face masks using catalytic pyrolysis with Ni–Fe bimetallic catalysts. The as-prepared CNTs from waste face masks displayed an SSA of 152.29 m^2^ g^–1^ and achieved a specific capacitance of 56.04 F g^–1^ in a 6 M KOH electrolyte at a 0.5 A g^–1^ current density. Similarly, Hu et al.^34^ synthesized high SSA (2220 m^2^ g^–1^) S-doped porous carbon from used face masks, which displayed a specific capacitance of 328.9 F g^–1^ at a current density of 1 A g^–1^ in a 6 M KOH electrolyte. Furthermore, the solid-state supercapacitor fabricated with a S-doped carbon electrode and a PVA/KOH electrolyte displayed an energy density of 10.4 W h kg^–1^ at a power density of 600 W kg^–1^.

However, the performance of activated carbon electrodes is limited by their narrow pore size and restricted ionic transport inside the porous medium. Hence, in order to address the drawbacks of activated carbon, researchers focused on integrating transition-metal sulfides with porous carbon carbons.^27^ For instance, nanoflower like MoS_2_ has been directly grown on corncob-derived activated carbon (CB-C) and utilized as electrodes for supercapacitors by Wang et al.^27^ The as-prepared MoS_2_/CB-C exhibited a specific capacitance of 333.5 F g^–1^ in a 1 M Na_2_SO_4_ electrolyte, and the fabricated symmetric supercapacitor possessed an energy density of 7.6 W h kg^–1^. Similarly, nickel sulfide/hemp-activated carbon (Ni_3_S_2_/3D HAC) composite has been successfully grown in a Ni-foam current collector using a hydrothermal technique by Shi et al.^35^ As reported by the authors, the Ni_3_S_4_/WHAC composite electrode exhibited a specific capacitance of 2797.43 F g^–1^ (0.7 V potential window) at a 1 A g^–1^ current density in the 6 M KOH electrolyte. All of the above results indicate that the addition of transition-metal sulfides to the porous activated carbon framework can transform the morphology of activated carbon and prevent the volume expansion/agglomeration of metal sulfide materials to a certain extent, resulting in enhanced electrochemical properties. In this regard, we carried out a simple hydrothermal technique for the first time to anchor tin sulfide material onto plastic waste (used face masks)-derived activated carbon.

Although few reports are available in the literature on screen-printed supercapacitors with activated carbon electrodes, there is no reported research about the utilization of face-mask-derived activated carbon/tin sulfide composites as electrodes for interdigitated stretchable supercapacitors. In this study, the used face masks were employed as the carbon source to prepare activated carbon (ACFM-T) via a high-temperature activation technique. The effect of the activation temperature on the textural properties of ACFM-T was also investigated. The hydrothermal method was later used to anchor tin sulfide (TS) on the high-surface-area activated carbon (ACFM-850), which led to the formation of a novel ACFM-850/TS composite. The novelty of this work comes from the ACFM-850/TS composite, which has not been explored before. The electrochemical performance of the as-prepared ACFM-850/TS composite was found to be impressive in a 1 M Na_2_SO_4_ electrolyte and proved it to be an efficient candidate as an electrode for stretchable supercapacitor fabrication. Furthermore, the screen-printing technique is employed to fabricate an interdigitated stretchable supercapacitors on Spandex fabric. The ACFM-850/TS composite-based stretchable supercapacitor exhibited high areal capacitance (33.8 mF cm^–2^ at 0.2 mA cm^–2^) and retained 64% of its performance after 10,000 cycles. The results of this work are very promising and pave the way for the possibility of using screen-printing technology to fabricate other plastic/biomass waste-derived carbon and its composite electroactive materials into interdigitated patterns on different substrates. Furthermore, this work can also be extended to fabricate asymmetric supercapacitors with plastic/biomass waste-activated carbon as one electrode and pseudocapacitive/conductive polymers as the other electrode. Additionally, screen printing is highly inexpensive and the entire process can be carried out under ambient conditions.

## 2 Experimental Section

### 2.1 Materials and Methods

#### 2.1.1 Materials and Reagents

Potassium hydroxide (KOH), sodium sulfate (Na_2_SO_4_), sodium perchlorate (NaClO_4_), poly(vinylidene fluoride) (PVDF) binder, 1-methylpyrrolidone, sulfuric acid (H_2_SO_4_), tin chloride (SnCl_2_), thiourea, and carboxymethyl cellulose were obtained from Sigma-Aldrich. Ethanol and hydrochloric acid were obtained from Fisher Scientific. Silver (Ag) conductive paste (PE872) was purchased from Dupont.

#### 2.1.2 Synthesis of Activated Carbon from Face Masks

Initially, disposable face masks were cut into small pieces. Then, 0.9 g of shredded face-mask pieces and 1.8 g of KOH were mixed with 40 mL of deionized (DI) water and stirred for 1 h. After 1 h, 1.5 mL of concn H_2_SO_4_ was added to the above mixture, which was then stirred for 2 h. After 3 h of stirring, the beaker was placed inside a hot air oven at 150 °C and dried for 90 h. After drying, the black product was calcined at 650–950 °C (heating rate: 10 °C min^–1^) for 2 h in the presence of nitrogen. After being cooled to room temperature, the activated samples were sufficiently washed with 1.0 M hydrochloric acid and distilled water several times, followed by drying at 80 °C overnight. The as-obtained samples were denoted as ACFM-T, where ACFM indicates activated carbon from the face mask and T indicates the activation temperature.

#### 2.1.3 Synthesis of Tin Sulfide/ACFM-850 Using the Hydrothermal Method

Briefly, 1 g of tin chloride dihydrate (SnCl_2_·2H_2_O) and 100 mg of ACFM-850 were mixed in 40 mL of DI water and stirred for 15 min on a magnetic stirrer. Afterward, 2 g of thiourea was added to the mixed solution and stirred for 1 h at room temperature. After 1 h, the black-colored solution was transferred into a 100 mL Teflon container and heated at 180 °C for 24 h. After cooling to ambient temperature, the precipitate was collected, washed with deionized water to eliminate contaminants, and dried in an oven at 80 °C overnight. For comparison, pure tin sulfide was prepared by a similar process without the addition of ACFM-850. The as-prepared samples were denoted as TS and ACFM-850/TS, where TS indicates tin sulfide.

### 2.2 Material Characterizations

X-ray diffraction (XRD) patterns were obtained by utilizing a Bruker D2 Phaser system with monochromatic Cu Kα radiation (α = 1.5406 Å). The samples were scanned with an increase of 0.04 on a scale from 5 to 80°. Throughout the measurements, the rotational speed of the substrates was kept at 8° min^–1^. The specific surface area (SSA) and pore volume of the as-prepared samples were determined using the Brunauer–Emmett–Teller (BET) and nonlinear density functional theory (NLDFT) methods on a Micrometrics-ASAP 2020 porosity analyzer. Prior to the analysis, all of the samples were degassed at 300 °C for 3 h in a dynamic vacuum. Raman spectra were obtained using a WiTec Raman microscope (excitation wavelength = 532 nm, laser power = 14.7 mW, acquisition time = 10 s, 10X objective lens) in the spectral region of 500–3500 cm^–1^. MATLAB script was used to average the five distinct spectra captured from different areas of the samples. Sample morphology was analyzed using an FEI Quanta 250 FEGSEM instrument with a 5 kV electron beam. High-angle annular dark-field scanning transmission electron microscopy (HAADF-STEM) measurements were performed on an FEI Titan Themis at 200 kV with a CEOS DCOR probe corrector, a SuperX energy dispersive X-ray spectrometer (EDX), and a 4k × 4k Ceta CMOS camera. A Thermo Scientific Kα X-ray photoelectron spectrometer (East Grinstead, U.K.) was utilized for X-ray photoemission spectroscopy (XPS) analysis. A hemispherical electron analyzer with a pass energy of 40 eV and a step size of 0.05 eV was used to obtain high-resolution photoemission spectra for specific elements. XPS spectra were captured using a monochromatic Al K_α_ X-ray source with a maximum beam spot size of 400 μm and an output energy of 1486.6 eV. A low-energy dual-beam electron/ion flood cannon was employed to modify the surface charge. The XPS and Raman spectra were all normalized before being deconvoluted by using Fityk software and Voigt fitting.

### 2.3 Electrode Preparation for Three-Electrode Measurements

For three-electrode measurements, a homogeneous slurry was prepared by mixing a 90:10 ratio of the electroactive material [ACFM-T, TS, ACFM-850/TS] and the PVDF binder with a few drops of 1-methylpyrrolidone. After that, the slurry was applied to a 1 cm^2^ area of a stainless-steel mesh (3 cm × 1 cm), and the electrodes were dried for 2 h at 80 °C. The wire diameter of the stainless-steel mesh was 0.025 mm, and its aperture measured 0.026 mm. The following was the weight of the activated carbon on the mesh electrodes: ACFM-650 = 2.5 mg, ACFM-750 = 2.4 mg, ACFM-850 = 2.1 mg, ACFM-950 = 2.2 mg, TS = 2.7 mg, and ACFM-850/TS = 2.1 mg.

### 2.4 Fabrication of a Stretchable Supercapacitor for Two-Electrode Measurements

In this study, a screen-printing process was employed to coat the electrode and the gel polymer electrolyte on the stretchable Spandex fabric. Initially, 40 mg of sodium carboxymethyl cellulose was dissolved in 10 mL of DI water, and the solution was stirred at 80 °C for 2 h. After cooling, 1 g of a finely ground ACFM-850/TS composite was added, and it was stirred for 2 h to form a slurry. In the second stage, the CMC/NaClO_4_ gel polymer electrolyte was prepared by mixing 2 g of carboxymethyl cellulose in 20 mL of DI water, and the solution was stirred for 1 h at 80 °C. After 1 h, 2 g of NaClO_4_ dissolved in 10 mL of DI water was added dropwise to the above mixture and stirred for another 1 h at 80 °C. After 2 h of vigorous stirring at 80 °C, a transparent thick gel was obtained, which was stored inside a desiccator for further use.

**Scheme 1:**
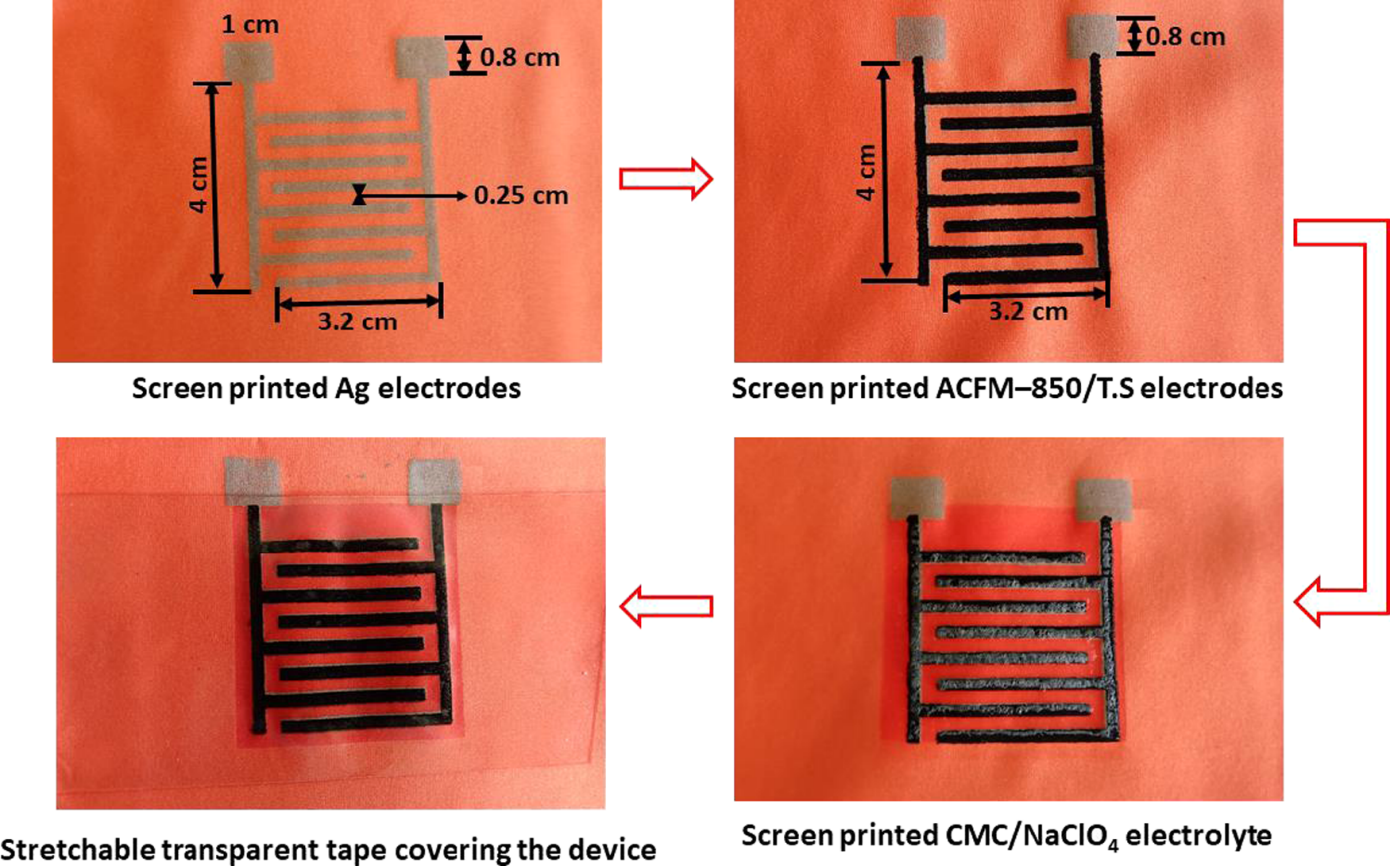
Photographs Showing Various Layers Screen-Printed for the Fabrication of a Supercapacitor on a Stretchable Spandex Fabric

Scheme 1 shows various layers screen-printed for the fabrication of stretchable supercapacitors on the Spandex fabric. For this purpose, Ag ink was first screen-printed on the Spandex fabric, followed by drying and annealing in an oven at 150 °C for 10 min. In the second step, the as-prepared ACFM-850/TS composite slurry was screen-printed on top of the Ag electrodes, followed by drying at 80 °C for 1 h in an oven. The CMC/NaClO_4_ gel electrolyte was then screen-printed on top to cover the electrode area, followed by degassing inside the desiccator for 1 h. Afterward, stretchable transparent tape was attached to the top of the device to prevent the electrolyte from drying.

### 2.5 Electrochemical Measurements

The electrochemical behavior of ACFM-T, TS, and ACFM-850/TS was investigated using an Autolab PGSTAT 302 N workstation. Ag/AgCl, a platinum wire, and an active material-coated stainless-steel mesh were utilized as the reference, counter, and working electrodes, respectively, for a standard three-electrode experiment. Next, 1.0 M Na_2_SO_4_ (1.0 M) was used in all of the above tests as the electrolyte. Galvanostatic charge–discharge (GCD) measurements were used to estimate the gravimetric specific capacitance (*C*_s_) of the electrode by equation by means of eq 1:^27,30,36^
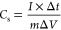
1where *m* is the mass of the active material on the electrode, Δ*t* is the discharge time after an IR drop, *I* is the current (A), and Δ*V* is the potential window during the discharge process.

Using galvanostatic charge–discharge (GCD) measurements, the areal specific capacitance (*C*_A_) (mF cm^–2^), energy density *E*_A_ (Wh cm^–2^), and power density *P*_A_ (W cm^–2^) for a stretchable supercapacitor in a two-electrode configuration were determined using eqs 2–4:^37,38^
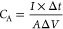
2
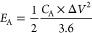
3
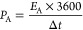
4where *S* is the area of the active material or the electrode covered with electrolyte, *I* is the discharge current, Δ*t* is the discharge period, and Δ*V* is the voltage window during the discharge process.

## 3 Results and Discussion

### 3.1 XRD Studies

**Figure 1:**
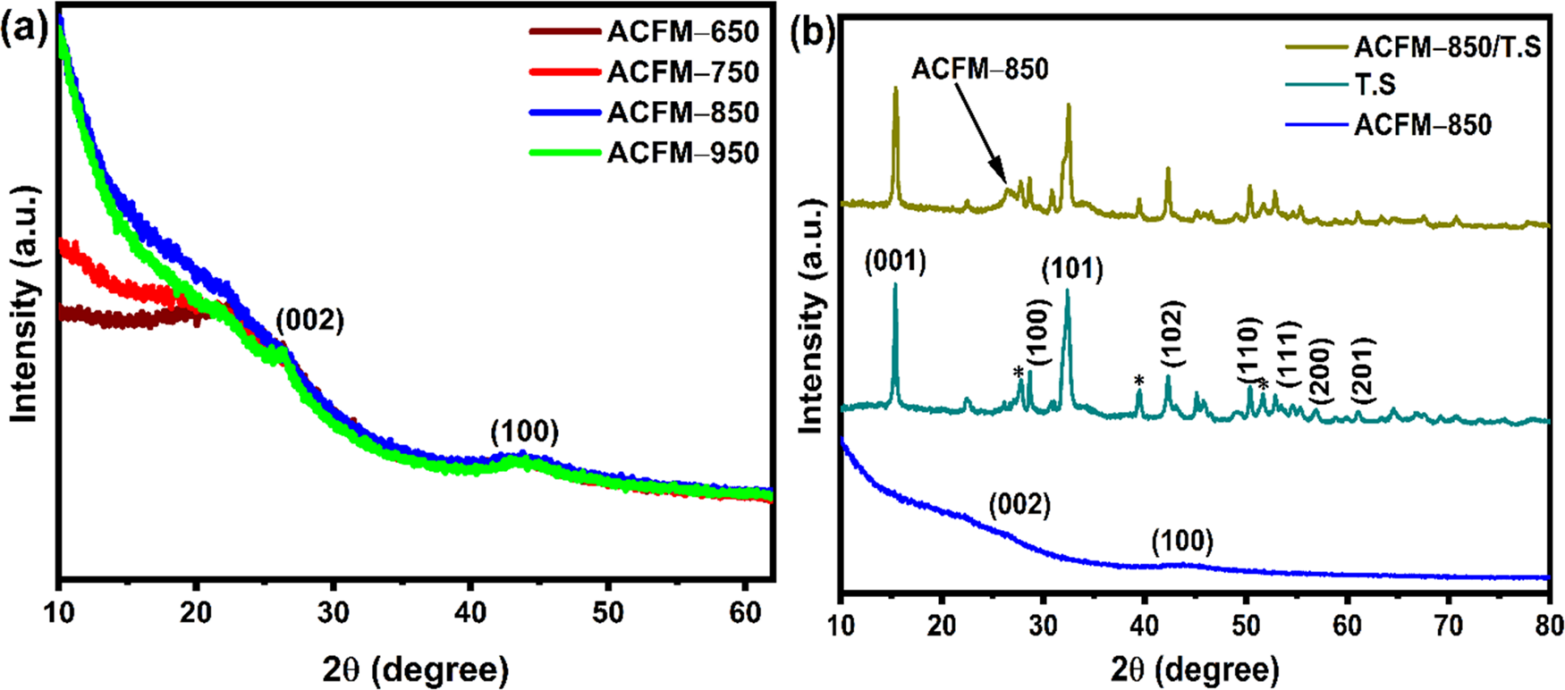
XRD patterns of (a) ACFM-T samples and (b) TS, ACFM-850, and ACFM-850/TS.

The crystalline phase and microstructure of the ACFM-T, TS, and ACFM-850/TS samples were verified using the XRD technique. The XRD patterns of each ACFM-T are comparable, as shown in Figure 1(a). Two peaks at 2θ = 26 and 44° were observed in each sample, and they are related to the (002) and (100) planes of amorphous carbon with graphitic structures.^30,39^ The high intensity in the low-angle region of ACFM-T indicates the presence of micropores in the samples.^40^Figure 1(b) shows the XRD patterns of tin sulfide (TS) and ACFM-850/TS composite samples. Pure tin sulfide (TS) shows diffraction peaks at 15.3, 28.7, 32.4, 42.1, 50.4, 52.8, 58.7, and 60.9°, which are attributed to the (001), (100), (101), (102), (110), (111), (200), and (201) characteristic planes of layered tin sulfide.^24,28^ The XRD pattern of tin sulfide shows a few more additional peaks at around 27, 38.5, and 51.7° indicated using “*”, which might correspond to the tin oxide phase.^41,42^ On the other hand, the XRD pattern of ACFM-850/TS shows a peak at around ∼26°, which is related to the (002) plane of activated carbon ACFM-850. Furthermore, all of the diffraction peaks of tin sulfide are visible in the ACFM-850/TS sample, indicating the successful formation of tin sulfide within the activated carbon framework.

### 3.2 N_2_ Adsorption/Desorption Studies

**Figure 2:**
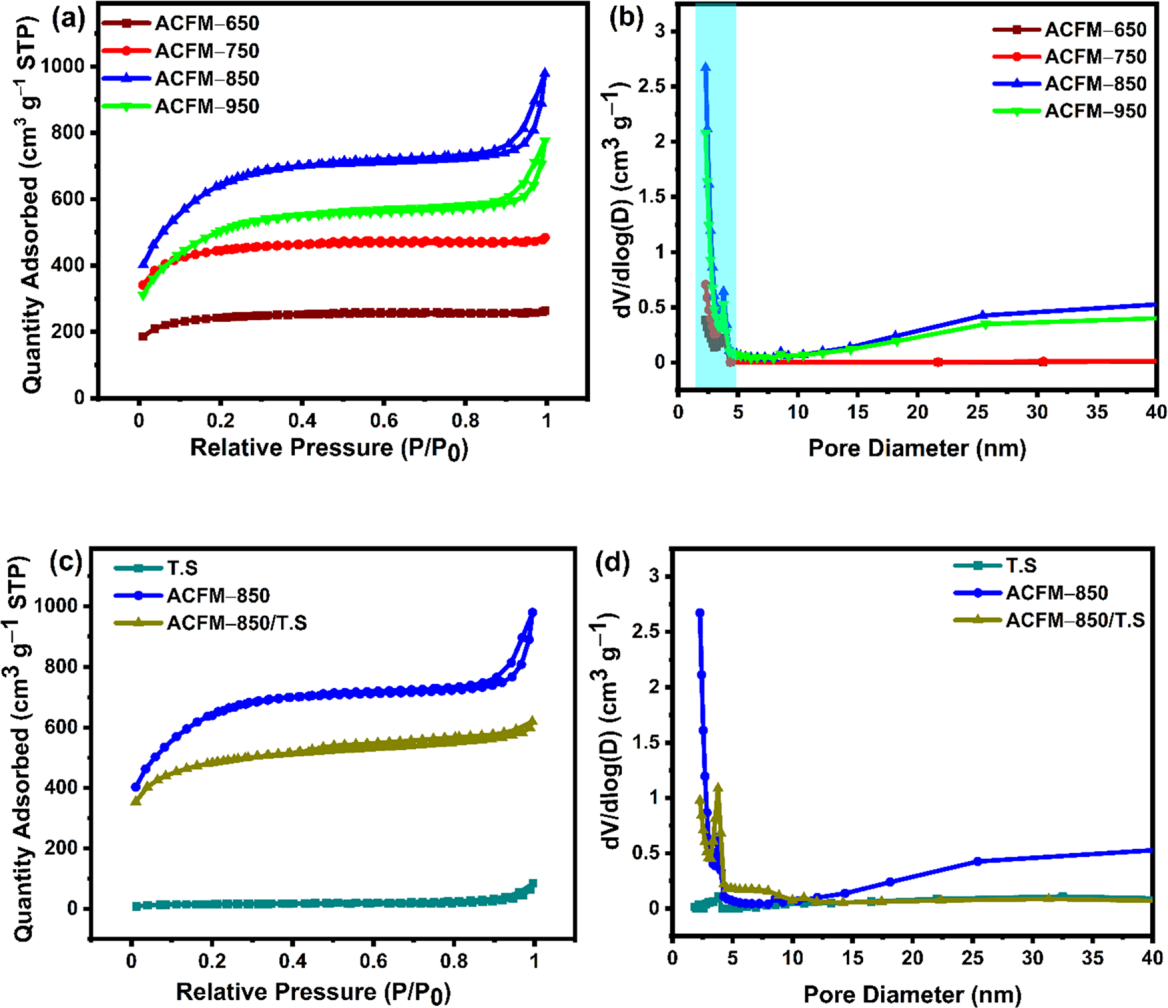
(a) N_2_ adsorption/desorption isotherms ACFM-T. (b) Pore size distribution (PSD) curves of ACFM-T samples. (c) Nitrogen adsorption/desorption isotherms of TS, ACFM-850, and ACFM-850/TS. (d) Pore size distribution (PSD) curves of TS, ACFM-850, and ACFM-850/TS samples.

N_2_ (nitrogen) adsorption/desorption isotherms were used to determine the physical characteristics of all ACFM-T samples, including their specific surface area (SSA) and pore volume, as shown in Figure 2(a) and (b), respectively. As illustrated in Figure 2(a), all of the isotherms belong to type I/IV curves according to IUPAC classification models, which indicates the existence of micro- and mesoporous structures. Each curve shows a distinctive H3 hysteresis loop, which is associated with the capillary condensation in the mesopores.^43−46^ While the steep adsorption at higher relative pressure values (0.9–1.0) indicates the presence of micro-, meso-, and/or macropores in the ACFM-T samples, the abrupt increase in the adsorption isotherm at low relative pressures, i.e., below 0.4 *P*/*P*_0_, is attributed to abundant micropores.^30,46^ The SSA and pore volume increase when the activation temperature increases from 650 to 850 °C, as indicated in Table 1. Increasing the activation temperature from 650 to 850 °C accelerates the release of volatiles in the precursor, which promotes the growth of existing pores and the creation of new pores. However, the SSA of the ACFMs is reduced from 2190 to 1702 m^2^ g^–1^ when further increasing the activation temperature from 850 to 950 °C due to the collapse in pore structure at a higher activation temperature of 950 °C. Furthermore, as shown in Figure 2(b), all of the samples possess mesopores with sizes between 2 and 5 nm, despite the fact that their pore volume varies drastically. The maximum pore volume of 1.53 cm^3^ g^–1^ was obtained for ACFM-850 activated at 850 °C, whereas ACFM-650 possesses the least pore volume (0.40 cm^3^ g^–1^). Hence, it can be concluded that the resulting ACFM-850 sample with high SSA and pore volume provides abundant pore structure for the growth of tin sulfide nanoparticles.

**Table 1:** SSA and Pore Volume of Different Samples

sample	SSA (m^2^ g^–1^)	pore volume (cm^3^ g^–1^)
ACFM-650	774	0.40
ACFM-750	1419	0.74
ACFM-850	2190	1.53
ACFM-950	1702	1.20
TS	43	0.10
ACFM-850/TS	1560	0.96

The N_2_ adsorption/desorption isotherms for TS, ACFM-850, and ACFM-850/TS samples are displayed in Figure 2(c), and the corresponding pore size distribution is shown in Figure 2(d). Clearly, ACFM-850, without the addition of tin sulfide nanoparticles, possesses the largest SSA of 2190 m^2^ g^–1^. The SSA of the composite integrated with tin sulfide nanoparticles decreases significantly, which is mainly attributed to the introduction of tin sulfide into the porous carbon framework where the ACFM-850 pores are occupied by tin sulfide. This is also evident in the pore size distribution curves exhibited in Figure 2(d), where the pore volume is reduced in the ACFM-850/TS composite. The ACFM-850/TS composite possesses a larger SSA of 1560 m^2^ g^–1^ and a pore volume of 0.96 cm^3^ g^–1^ than pure tin sulfide (SSA = 43 m2 g^–1^, pore volume = 0.10 cm^3^ g^–1^). The presence of tin sulfide in the composite exposes more surface active sites and provides easy access to speed up the transport and diffusion of electrolyte ions inside the porous ACFM-850 network. Hence, ACFM-850/TS is expected to provide enhanced electrochemical performance compared to pure ACFM-850 and TS.

### 3.3 Raman Studies

**Figure 3:**
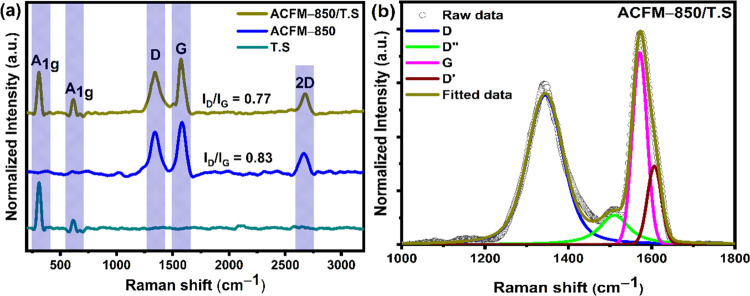
(a) Raman spectra of TS, ACFM-850, and ACFM-850/TS samples. (b) Deconvoluted Raman spectra of the ACFM-850/TS sample.

Figure 3(a) displays the Raman spectra of TS and ACFM-850 and ACFM-850/TS samples, and Figure S1a (Supporting Information) shows the Raman spectra of the remaining ACFM-T samples. As shown in Figure S1a, ACFM-T samples possess two strong peaks centered at 1345 ± 5 and 1576 ± 5 cm^–1^, respectively. These peaks belong to D bands, which are assigned to disordered carbon atoms, and G bands, which are attributed to the sp^2^-hybridized graphitic carbon atoms, respectively.^47^ The Raman spectra of ACFM-T samples are further deconvoluted into four distinct peaks, as shown in Figure S1b–e. According to the literature, the peaks centered at around 1240, 1347, 1487, and 1580 cm^–1^ are attributed to the D* band, D band, D″ band, and G band, respectively. In general, the D* band corresponds to the polyene/oligomer, the D band arises from the disorder/defects in the lattice structure of the carbon materials, the D″-band represents the amorphous carbon structure, and the G band is attributed to the sp^2^-hybridized graphitic carbons embedded in the porous carbon framework.^30,48,49^ The intensity ratio of the D and G band (*I*_D_/*I*_G_) reflects the degree of graphitization in the carbon materials.^48^ As shown in Figure S1a, the ACFM-850 sample exhibited the lowest *I*_D_/*I*_G_ value, which corresponds to the highest degree of graphitization among all of the face-mask-derived activated carbons. From the Raman and N_2_ sorption analysis, it can be inferred that the ACFM-850 sample possesses a higher degree of graphitization and high SSA, where the high SSA provides more surface active sites for the electrolyte ions to access, whereas the higher graphitization degree promotes the charge-transport kinetics and electrolyte ion penetration into deeper regions of the porous electrode framework. Hence, the optimized ACFM-850 sample was utilized for preparing the composite because of its excellent textural characteristics compared to the other face-mask-derived activated carbons.

For the as-prepared tin sulfide (TS) material, a strong and sharp band at ∼310 cm^–1^ is attributed to the A_1g_ vertical-plane vibrational mode of the Sn–S bonds, demonstrating the presence of tin sulfide.^26^ Raman spectra of tin sulfide also consist of a low-intensity peak detected at 615 cm^–1^ corresponding to the A_1g_ mode of Sn–O bonds, indicating a tiny amount of tin oxide in the sample.^50^ The Raman spectra of the ACFM-850/TS composite consist of D and G bands besides the tin sulfide peaks (at ∼310 and 615 cm^–1^), indicating the coexistence of tin sulfide (TS) and activated carbon (ACFM-850), which is consistent with XRD data shown in Figure 1(b). Figures 3(b) and S1a–e show the Raman spectra of the acquired samples, which are all convolved into four peaks. The D and G peaks of the ACFM-850/TS sample are further deconvoluted into four separate bands ascribed to the disordered amorphous structure (D band, at 1340 cm^–1^), amorphous carbon (D″ band, at 1485 cm^–1^), graphitic carbon (G band, at 1571 cm^–1^), and D′ band (1606 cm^–1^) respectively.^30,48,51^ The degree of graphitization in the synthesized samples is determined by the relative intensity ratio (*I*_D_/*I*_G_) of the D band to the G band; the smaller the *I*_D_/*I*_G_ ratio, the more graphitized the carbon material.^48,52^ The *I*_D_/*I*_G_ ratio of the ACFM-850/TS composite was calculated to be 0.77, which is lower compared to ACFM-850 (0.83), indicating that the ACFM-850/TS composite may have a more efficient charge-transfer process than other samples.

### 3.4 XPS Analysis

**Figure 4:**
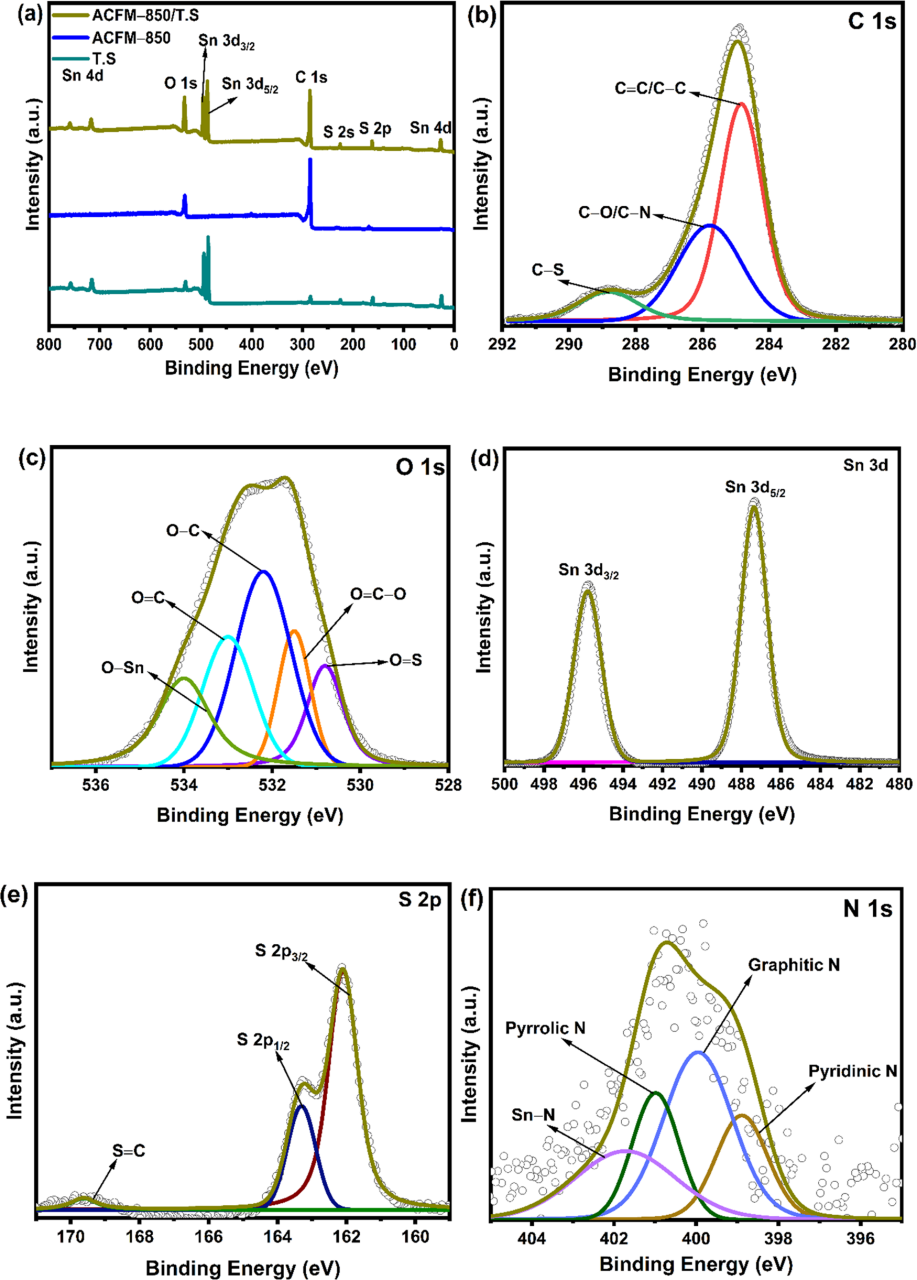
(a) XPS survey spectra of TS, ACFM-850, and ACFM-850/TS samples; high-resolution deconvoluted XPS spectra of (b) C 1s, (c) O 1s, (d) Sn 3d, (e) S 2p, and (f) N 1s.

The surface bonding of the TS, ACFM-T, and ACFM-850/TS is further studied by XPS analysis, and the spectra are shown in Figures 4(a) and S3a. The XPS survey spectra of the ACFM-850/TS displayed in Figure 4(a) reveal clearly defined peaks at 165 eV (S 2p), 285.0 eV (C 1s), 400 eV (N 1s), 487 eV (Sn 3d), and 531 eV (O 1s) eV, respectively.^24^ The distinct peaks at 24, 717, and 758 eV are characteristic of the Sn 4d state, whereas the peak at 226 eV arises from the S 2s state of tin sulfide material embedded in a porous carbon framework.^24,26^ The XPS survey spectra of the ACFM-850/TS composite confirm the coexistence of tin sulfide (TS) and activated carbon (ACFM-850) samples. In the survey spectra of the ACFM-850/TS composite, the intensity of C 1s and N 1s is much lower than that of ACFM-850, showing that tin sulfide has been successfully deposited on the surface of ACFM-850, which is compatible with the XRD and Raman results. The C 1s spectrum [Figure 4(b)] of ACFM-850/TS can be deconvoluted into three main peaks located, respectively, at 284.5 (C=C/C–C), 286 (C–O/C–N), 287.5 (C–S), and 289 (C=O) eV.^52,53^ As shown in Figure 4(c), five peaks centered at around 531, 531.8, 532.6, 533.3, and 534 eV of the O 1s spectrum correspond to O–Sn, O=C, O–C, O=C–O, and O=S, respectively, which are mainly attributed to the oxygen functionalities in the ACFM-850/TS composite.^24,30^ N 1s spectra [Figure 4(f)] are deconvoluted into four peaks: 398.7 (pyridinic N), 399.7 (graphitic N), 400.3 (pyrrolic N), and 401.2 (Sn–N, specifies the interaction between tin sulfide and the N atom of ACFM-850).^24^ The high-resolution Sn 3d peaks shown in Figure 4(d) are further deconvoluted into two distinct peaks positioned at 487.2 and 495.7, eV corresponding to Sn 3d_5/2_ and Sn 3d_3/2_ states, respectively, which designates the presence of the Sn^4+^ oxidation state of tin in the composite.^24^ From the S 2p spectra in Figure 4(e), the fitted peaks at 162.1, 163.4, and 168.6 eV are assigned to S 2p_3/2_, S 2p_1/2_, and S=C, respectively, indicating multiple combinations of the sulfur element on the surface of the ACFM-850/TS composite framework. The XPS findings imply that the ACFM-850/TS composite has been successfully synthesized, which agrees with the XRD [Figure 1(b)] and Raman analyses [Figure 3(a)].

### 3.5 Morphological Analysis

**Figure 5:**
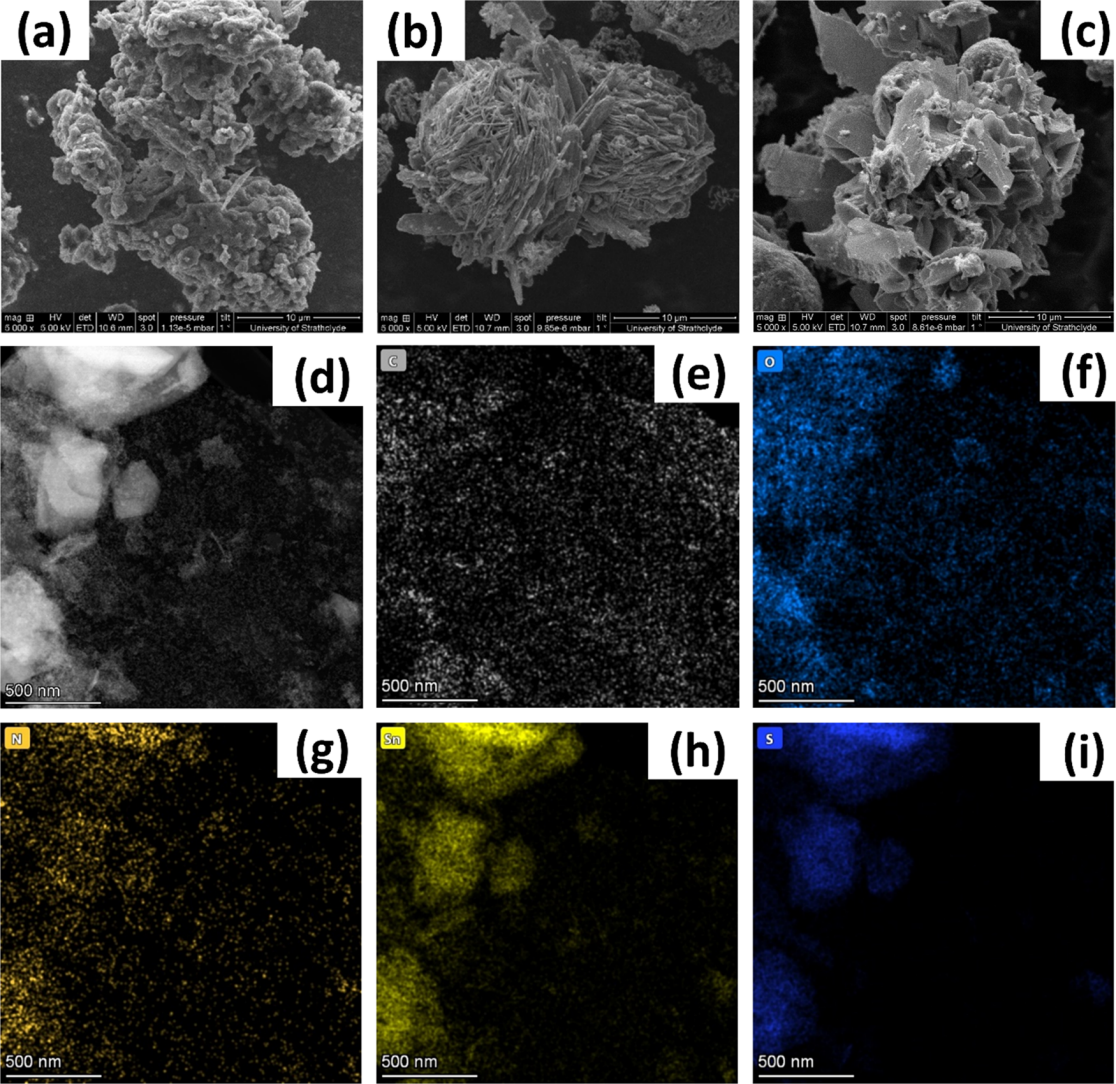
FESEM images of as-prepared (a) ACFM-850, (b) tin sulfide (TS), and (c) ACFM-850/TS samples. (d) HAADF-STEM image of the ACFM-850/TS composite with a scale bar of 500 nm. (e–i) EDS elemental mapping of C, O, N, Sn, and S elements of the ACFM-850/TS composite.

**Figure 6:**
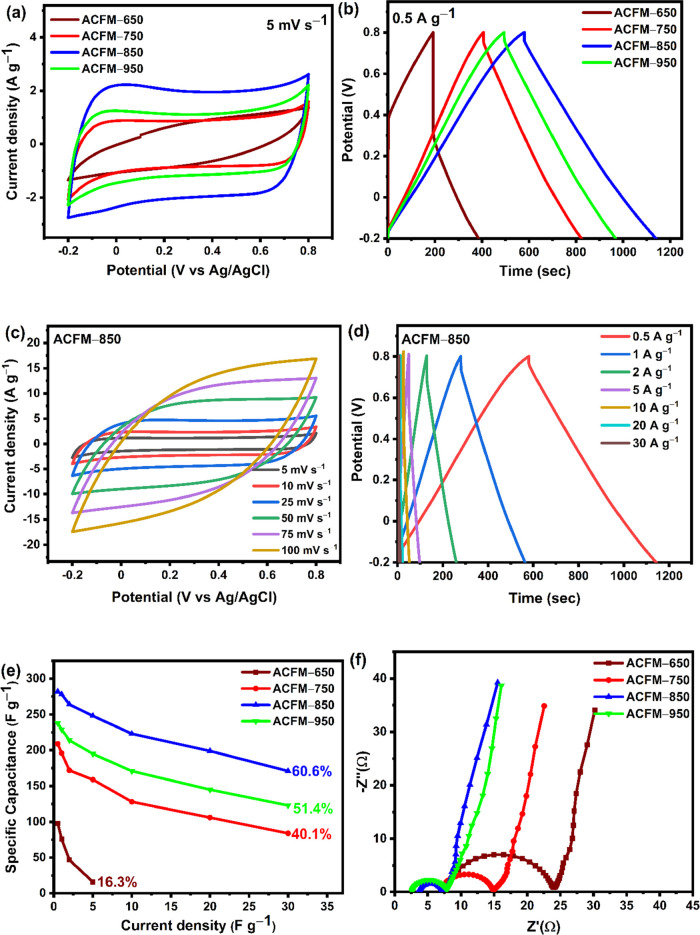
Electrochemical performance of the ACFM-T electrodes in a 1 M Na_2_SO_4_ electrolyte: (a) cyclic voltammetry (CV) curves of ACFM-T electrodes at a scan rate of 5 mV s^–1^. (b) GCD graphs of ACFM-T electrodes at a current density of 0.5 A g^–1^. (c) CV curves of the ACFM-850 electrode at various scan rates. (d) GCD curves of the ACFM-850 electrode at various current densities. (e) Specific capacitance of ACFM-T electrodes plotted as a function of different current densities. (f) Nyquist plots for all of the ACFM-T electrodes measured in a three-electrode system.

**Figure 7:**
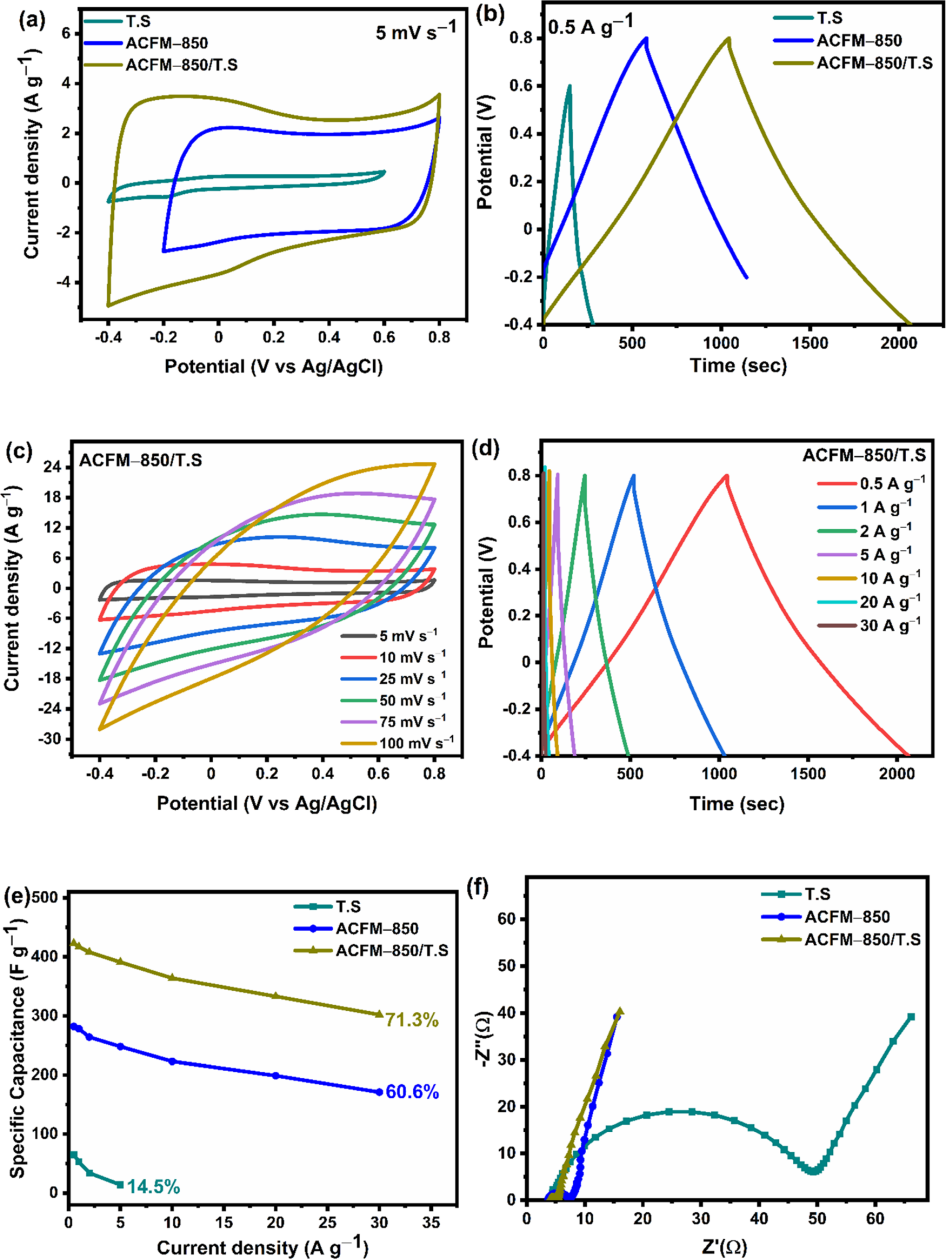
Electrochemical performance of the TS, ACFM-850, and ACFM-850/TS electrodes in a 1 M Na_2_SO_4_ electrolyte. (a) CV graphs of TS, ACFM-850, and ACFM-850/TS electrodes at a 5 mV s^–1^ scan rate. (b) GCD curves of TS, ACFM-850, and ACFM-850/TS electrodes at a 0.5 A g^–1^ current density. (c) CV curves of the ACFM-850/TS electrode at different scan rates. (d) GCD curves of the ACFM-850/TS electrode at different current densities. (e) Specific capacitance of TS, ACFM-850, and ACFM-850/TS electrodes plotted as a function of different current densities. (f) Nyquist plots of TS, ACFM-850, and ACFM-850/TS measured in a three-electrode system.

Figure 5 shows the field-emission scanning electron microscopy (FESEM) images showing the morphology of the synthesized TS, ACFM-850, and ACFM-850/TS composites. According to Figure 5(a), ACFM-850 exhibits a hierarchical porous structure consisting of a rough and uneven surface with irregular craters and pores. The porous nature of the ACFM-850 sample was confirmed by the high-resolution transmission electron microscopy (HRTEM) measurements shown in Figure S2a,b. The HRTEM images of the ACFM-850 sample clearly indicate the existence of amorphous porous carbons with ultrathin graphitic carbon layers accumulated in the carbon framework. The porous structure, along with large SSA, provides sufficient channels for the migration and storage of electrolyte ions, consequently ensuring excellent electrochemical performance of the ACFM-850 sample, as shown in Figure 6. However, we believe that these porous structures gradually aggregate, which reduces the exposure of surface active sites for the adsorption/desorption of ions. Pure tin sulfide materials exhibit a spherical ball-like morphology, aggregated in some regions, with each ball consisting of well-defined, discrete sheet-, or plate-like structures, as shown in Figure 5(b). Although the sheet-like structures in spherical tin sulfide allow deep electrolyte penetration, they tend to restack during the adsorption/desorption process, resulting in poor transportation of electrolyte ions, which can explain its inferior electrochemical performance, as discussed in Figure 7. Upon compositing ACFM-850 with tin sulfide, it can be seen that tin sulfide sheets randomly grew on the surface and inside the conductive porous ACFM-850 matrix, as shown in Figure 5(c). Furthermore, HAADF-STEM images of the ACFM-850/TS composite shown in Figure 5(d) provided clear evidence of the coating of tin sulfide (TS) in porous ACFM-850. In addition, the elemental distribution also revealed the presence of C, O, N, Sn, and S elements in the ACFM-850/TS composite, as shown in Figure 5(e–i). We believe, during a hydrothermal reaction, ACFM-850 acts as a template to aid the growth of tin sulfide sheets and develops into a well-interconnected architecture, which not only prevents the agglomeration of tin sulfide sheets but also favors the maximum utilization of a porous ACFM-850 matrix. Furthermore, the tin sulfide sheets can prevent the agglomeration of the as-prepared porous carbon particles, which is essential during for the continuous charge–discharge cycles. This hierarchical porous architecture provides rapid ionic diffusion channels, which may result in more effective interaction between the ions and the active materials and potentially improve the electrochemical performance.

### 3.6 Electrochemical Analysis of ACFM-T Samples in a 1 M Na_2_SO_4_ Electrolyte Tested in a Three-Electrode System

The electrochemical performances of the activated carbons produced from face masks were initially examined in a three-electrode system with 1 M Na_2_SO_4_ as an electrolyte in order to identify the best-performing ACFM-T samples for composite synthesis. Figure 6(a) presents the results of the cyclic voltammetry (CV) graph of ACFM-T samples measured at a scan rate of 5 mV s^–1^ in the potential window of −0.2 to +0.8 V vs Ag/AgCl (Δ*V* = 1 V). The cyclic voltammogram plots of ACFM-T samples at a 5 mV s^–1^ scan rate resemble almost rectangular shapes, indicating the capacitive nature of the carbon material. From Figure 6(a), it can be inferred that the performance (current density) of activated carbon increases with the increase in activation temperature from 650 to 850 °C, which can be attributed to the increase in SSA from 774 to 2190 m^2^ g^–1^, indicating that a greater number of electrolyte ions can be stored in the electrode pores in ACFM-850, and thus results in a higher specific capacitance. Increasing the activation temperature to 950 °C (ACFM-950) decreased the performance of activated carbon, which can correspond to the decreased SSA and pore volume of the ACFM-950 sample (as shown in Table 1) as compared to the ACFM-850 sample because a high activation temperature causes the porosity to collapse. From Figure 6(a), it can be concluded that the ACFM-850 sample possesses the highest current density and the largest loop area, implying the highest specific capacitance, and is the best candidate for the formation of composite material. The GCD curves of the ACFM-T samples [Figure 6(b)] show almost linear charge–discharge characteristics, which is typical of carbonaceous materials. Interestingly, the ACFM-850 electrode exhibits the longest charge/discharge duration at a 0.5 A g^–1^ current density, indicating that a greater number of pores are accessed by the electrolyte ions, which is attributed to its high SSA, pore volume, and greater degree of graphitization of ACFM-850 samples compared to other samples. At a 0.5 A g^–1^ current density, the specific capacitance of ACFM-T samples is in the order of ACFM-850 (282 F g^–1^) > ACFM-950 (238 F g^–1^) > ACFM-750 (209 F g^–1^) > ACFM-650 (97 F g^–1^), respectively, with ACFM-850 achieving the highest performance and ACFM-650 being the lowest. The specific capacitance of activated carbon samples generally increases with the increase in SSA of the samples, and in this work, ACFM-850 showed the highest SSA, resulting in high specific capacitance.^30,31^ From Figure 6(c), it can be observed that the CV curves appear rectangular at lower scan rates (5–25 mV s^–1^) and quasi-rectangular at higher scan rates (50–100 mV s^–1^), indicating better electrolyte ion adsorption and desorption on the electrode surface at lower scan rates. At the highest scan rate of 100 mV s^–1^, the curves transform into an eye-like shape, which indicates restricted ionic transfer and a decrease in capacitive behavior. The deviation in the capacitive behavior at higher scan rates could be related to the existence of functional groups on the surface of the electrode materials, which produce tiny amount of pseudocapacitance or impacted by the ohmic resistance of the electrode.^54^ The charge–discharge behavior of ACFM-850 is shown in Figure 6(d) at current densities ranging from 0.5 to 30 A g^–1^. The ACFM-850 electrode retains triangular-shaped curves even at elevated current densities of 30 A g^–1^, further demonstrating the excellent rate capability and electrochemical reversibility of ACFM-850 electrodes. Figure 6(e) presents the specific capacitance of the ACFM-T electrodes plotted as a function of the current density. As shown in Figure 6(e), ACFM-850 samples retain 60.6% of their initial capacitance even at a high current density of 30 A g^–1^, which can be explained by their hierarchically porous framework with a high graphitization degree, SSA, and pore volume.

Figure 6(f) shows the Nyquist plot obtained from EIS in the frequency range of 10 mHz–100 kHz and an amplitude of 5 mV. For the ACFM-850 electrode, the vertical line in the low-frequency zone is more nearly parallel to the imaginary axis, which indicates the nearly perfect capacitive behavior of the ACFM-850 electrode. Conversely, for samples ACFM-650, ACFM-750, and ACFM-950, the vertical lines sloped away from the imaginary axis, suggesting poor capacitive behavior because of the decreased SSA and pore volume. The equivalent series resistance (*R*_s_), which is the sum of the contact resistance with the current collector, the interfacial resistance of the electrode material, and the ionic resistance of the electrolyte, is often estimated using the intercept of the Nyquist plot with the *x*-axis.^55^ The diameter of the semicircle in the intermediate- to high-frequency range indicates the electrode’s charge-transfer resistance (*R*_ct_). As shown in Figure 6(e), as the activation temperature increases from 650 to 850 °C, the charge-transfer resistance (diameter of the semicircle) decreases. This is due to an increase in the graphitization degree that creates more charge-transport pathways for the quick transfer of electrolyte ions within the porous structure of ACFM-850. A further increase in the activation temperature from 850 to 950 °C increases the *R*_ct_ value from 3.6 to 5.2 Ω, which might be due to the decrease in SSA and pore volume at higher activation temperatures. Electrochemical analysis of ACFM-T samples tested in a 1 M Na_2_SO_4_ electrolyte shows that the activation temperature has a significant influence on the capacitive performance of the electrode materials. The ACFM-850 sample demonstrated excellent electrochemical behavior with high specific capacitance, rate capability, and lower charge transfer, which is attributed to its hierarchical pore structure, larger (SSA), large pore volume (as shown in Table 1), and a higher degree of graphitization (as shown in Figure S1a) compared to other face-mask-derived activated carbons. Hence, ACFM-850 is employed as a template for growing tin sulfide inside the porous carbon network.

### 3.7 Electrochemical Analysis of TS, ACFM-850, and ACFM-850/TS Samples in a 1 M Na_2_SO_4_ Electrolyte Tested in a Three-Electrode System

Figure 7(a) shows the CV graphs of TS, ACFM-850, and ACFM-850/TS electrodes at a 5 mV s^–1^ scan rate in a 1 M Na_2_SO_4_ electrolyte. The CV profile for a sheet-like spherical tin sulfide electrode within −0.4 to 0.6 V vs Ag/AgCl reveals a weak curve, which indicates the lowest electrochemical performance. When compared to pure tin sulfide and ACFM-850 electrodes, the ACFM-850/TS composite showed a rectangular CV curve with improved current density and potential window (−0.4 to +0.8 V = 1.2 V vs Ag/AgCl), which is likely due to the combined effect of the tin sulfide and ACFM-850 components in the ACFM-850/TS composite. GCD tests were carried out to evaluate the potential of the pure TS, ACFM-850, and composite ACFM-850/TS as an electrode material for supercapacitors. As illustrated in Figure 7(b), at the same current density of 0.5 A g^–1^, all of the electrodes displayed a nearly symmetric charging/discharging phenomena with the ACFM-850/TS composite occupying longer charge/discharge times than compared to individual TS and ACFM-850 electrodes. This shows that the composite surface is used more extensively for the adsorption/desorption of electrolyte ions, thereby enhancing the electrochemical behavior than its individual counterparts. Figure 7(c) characterizes the CV curves of ACFM-850/TS at various scan rates from 5 to 100 mV s^–1^, where the CV curves remain rectangular until a 25 mV s^–1^ scan rate, after which they begin to distort. This suggests that the electrolyte ions may readily contact the electrode surface for a long time and that the ionic movement is fast enough at lower scan rates, resulting in capacitive-type behavior. Higher scan rates, on the other hand, limit the flow of electrolyte ions due to insufficient reaction time and enhance charge diffusion polarization inside electrode materials, thereby reducing the performance of the electrode material.^56,57^Figure 7(d) demonstrates the charge/discharge characteristics of the ACFM-850/TS composite electrode at different current densities (0.5–30 A g^–1^) in 1 M Na_2_SO_4_ aqueous electrolyte. It can be noted that the duration of charging/discharging grows progressively as the current density decreases, which is attributable to sufficient insertion or release of electrolyte ions during the charging and discharging stages. Nonetheless, the ACFM-850/TS composite electrode retains its triangular-shaped curves even at higher current densities, demonstrating its reversible adsorption/desorption of ions during the charge/discharge process. Figure 7(e) displays the specific capacitance plotted against the current density for TS, ACFM-850, and ACFM-850/TS composite electrodes, as shown in Figure 7(e). According to Figure 7(e), the specific capacitance continues to decrease with increasing current densities because the inner active regions are unable to take part in the electrochemical process due to the shortened reaction time. With the addition of tin sulfide sheets to ACFM-850, the highest capacitance of 423 F g^–1^ was obtained for the ACFM-850/TS composite, surpassing that of pure tin sulfide (65 F g^–1^) and ACFM-850 (282 F g^–1^) electrodes at a current density of 0.5 A g^–1^. Meanwhile, the specific capacitance of ACFM-850/TS composite electrodes drops from 423 to 302 F g^–1^ (71.3% capacitance retention) with the increase in current density from 0.5 to 30 A g^–1^, which is superior to that of pure tin sulfide (14.5%) and pure ACFM-850 (60.6%) electrodes. These findings indicate that the synthesized ACFM-850/TS composite electrode has superior rate capability when compared to pure tin sulfide and ACFM-850 electrodes. Figure 7(f) shows the Nyquist plots of TS, ACFM-850, and ACFM-850/TS electrodes, which form a semicircle at the high-frequency zone and a nearly straight line at the lower frequencies. Their *R*_ct_ value, derived from the diameter of the high-frequency arc on the real axis, is different and follows the order: ACFM-850/TS (1.2 Ω) < ACFM-850 (3.6 Ω) < TS (42.5 Ω). Moreover, the smaller *R*_ct_ and a more vertical line along the imaginary axis for the ACFM-850/TS composite indicate the lower charge-transfer resistance and better capacitive behavior. These findings suggest that the ACFM-850/TS composite with tin sulfide sheets not only provides abundant conductive pathways for the diffusion of ions from the electrolyte to the porous carbon matrix but also improves the utilization of ACFM-850, endowing the ACFM-850/TS composite electrode with remarkable rate performance and high capacitance behavior.

Table 2 shows that the electrochemical performance of the ACFM-850/TS composite electrode is comparable to or better than the other biomass activated carbon/metal sulfide electrodes reported in the literature. Hu et al.^34^ synthesized high SSA (2220 m^2^ g^–1^) activated carbon (CMS-3) from waste polypropylene face masks, which showed a specific capacitance of 328.9 at 1 A g^–1^ in a 6 M KOH electrolyte. It can be observed that the performance of the CMS-3 electrode reported by Hu et al.^34^ is less than the values reported in this work for the ACFM-850/TS composite, which can be attributed to the presence of tin sulfide structures in the porous carbon matrix. On the other hand, the specific capacitance of the ACFM-850/TS composite tested in a 1 M Na_2_SO_4_ electrolyte is higher than the various biomass activated carbon/molybdenum composites (MoS_2_) reported in the literature.^27,58−60^ This can be attributed to the high SSA of the ACFM-850/TS composite, which resulted in a larger accumulation of electrolyte ions inside the composite framework. In contrast, the performance of the ACFM-850/TS composite electrode is less than the NiS/AC and CSPC-2 composite electrodes reported by Yang et al.^61^ and Li et al.^56^ Here, the important point to be noted is the operational potential windows of the electrodes. Although, NiS/AC and CSPC-2 composite electrodes exhibited higher specific capacitance, their operating potential is much less (<0.8 V) compared to the ACFM-850/TS composite electrode (1.2 V) reported in this work. The superior electrochemical performance can be mainly attributed to the hierarchical porous architecture of the ACFM-850/TS composite [shown in Figure 5(c)] with a synergistic effect between tin sulfide sheets and the porous ACFM-850 matrix, where the tin sulfide sheets not only act as an active electroactive material in the electrochemical process but also provide additional charge-transport channels for insertion and extraction of electrolyte ions inside the porous carbon matrix.

**Table 2:** Comparison of Electrochemical Performance (Specific Capacitance) of the ACFM-850/TS Composite Electrode with Other Biomass Activated Carbon/Metal Sulfide Composite Electrodes Reported in the Literature

electrode material	SSA (m^2^ g^–1^)	electrolyte	specific capacitance (F g^–1^)	ref
CMS-3 (face mask)	2220	6 M KOH	328.9 at 1 A g^–1^ (0.8 V)	(34)
PCS-MnO_2_ (PET)	453	6 M KOH	210 at 0.5 A g^–1^ (1.0 V)	(62)
MoS_2_/HPGC (pomelo peel)	320.2	3 M KOH	411 at 0.5 A g^–1^ (0.7 V)	(60)
NiS/AC (walnut shells)		2 M KOH	1688.5 at 1 A g^–1^ (0.5 V)	(61)
ACZS (waste engine oil)	1020	0.5 M H_2_SO_4_	241 at 1 A g^–1^ (1.1 V)	(63)
CSPC-2 (pomelo peel)	17.09	6 M KOH	954 at 1 A g^–1^ (0.7 V)	(56)
MoS_2_/CSAC	113.7	6 M KOH	332.6 at 1 A g^–1^ (0.4 V)	(58)
MoS_2_/CB-C (corn cob)	101	1 M Na_2_SO_4_	333.5 at 1 A g^–1^ (0.8 V)	(27)
K-MoS_2_ (kapok fiber)		1 M Na_2_SO_4_	254 at 0.5 A g^–1^ (1.0 V)	(59)
ACFM-850/TS	1560	1 M Na_2_SO_4_	423 at 0.5 A g^–1^ (1.2 V)	this work
			421 at 1 A g^–1^ (1.2 V)	

### 3.8 Electrochemical Performances of the Screen-Printed ACFM-850/TS-Based Symmetric Supercapacitor Fabricated on a Stretchable Spandex Fabric

**Figure 8:**
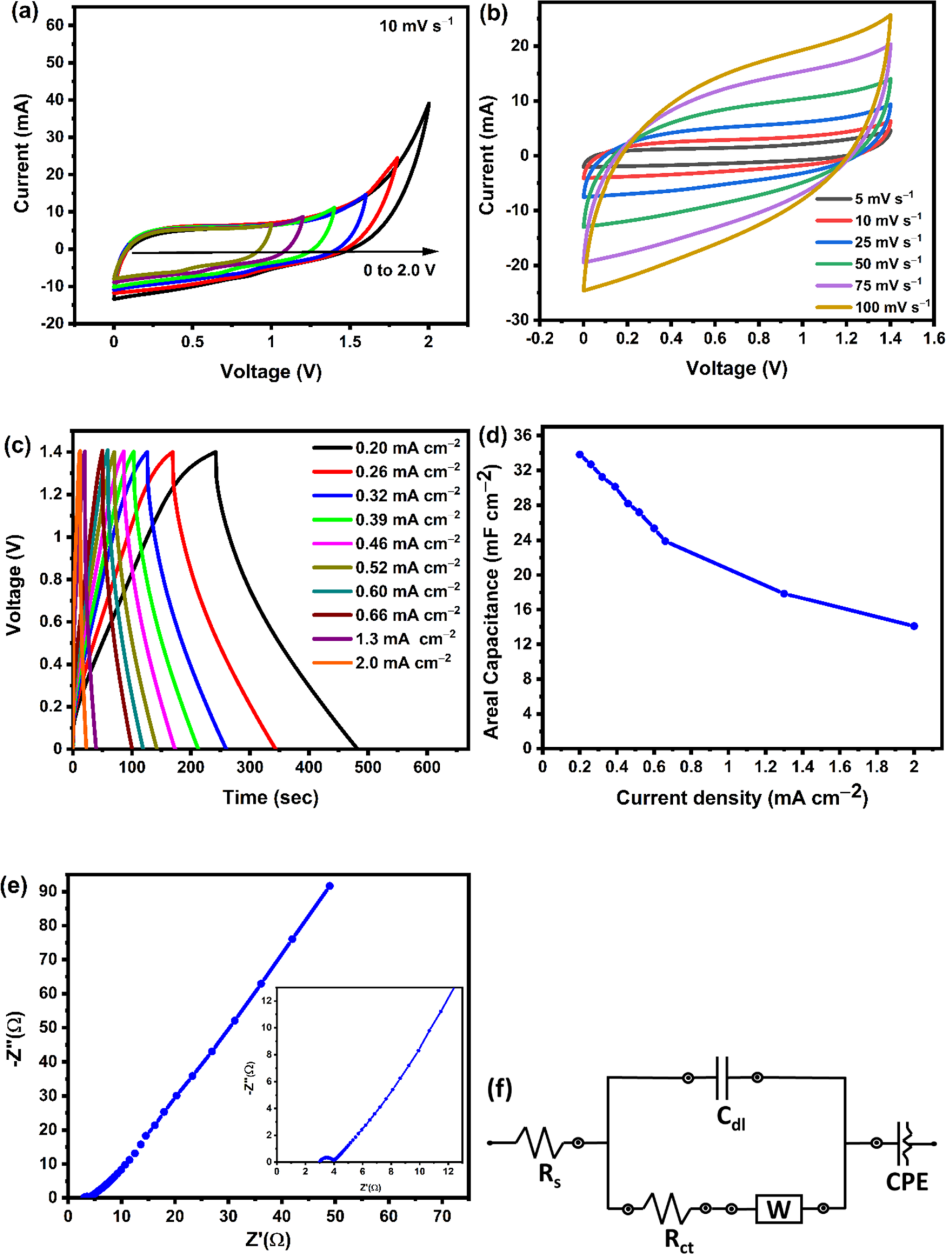
Electrochemical performances of the screen-printed ACFM-850/TS-based symmetric supercapacitor: (a) CV curves at various potential windows at a scan rate of 10 mV s^–1^. (b) CV curves at different scan rates from 5 to 100 mV s^–1^. (c) GCD plot at different current densities. (d) Areal capacitance of the supercapacitor plotted against current density. (e) Nyquist plot. (f) Equivalent circuit fitting model for the impedance spectrum of the symmetric supercapacitor.

The flexible supercapacitor based on the ACFM-850/TS composite was fabricated via screen printing to study the practical application, as shown in Scheme 1, where a stretchable Spandex fabric was used as a substrate for a screen-printing ACFM-850/TS composite slurry on a silver current collector with an interdigitated electrode pattern. It can be observed from the design that two electrodes were in planar geometry, and CMC/NaClO_4_ gel was employed as the ionic conducting medium between the two electrodes. Figure 8(a) shows the CV curves at various voltage ranges measured at a 10 mV s^–1^ scan rate to determine the maximum stable operating voltage of this supercapacitor. In this scenario, no obvious increase in current has been observed until 1.4 V, which indicates that this screen-printed supercapacitor device can be operated up to a voltage of 1.4 V, and beyond 1.4 V, there is a rapid increase in the current response specifying the decomposition of the gel electrolyte. This has been further verified from the charge–discharge measurements shown in Figure S7a (Supporting Information). As indicated in Figure S7a (Supporting Information), the supercapacitor exhibited a nearly linear charging curve until the 1.4 V potential window, beyond which a plateau can be observed in the graph, indicating electrolyte decomposition beyond 1.4 V. Therefore, a potential window of 1.4 V is chosen for safe operation of this device and further electrochemical investigations have been carried out. Figure 8(b) demonstrates that this screen-printed ACFM-850/TS-based symmetric supercapacitor evaluated at various scan rates between 5 and 100 mV^–1^ within the selected voltage range of 0–1.4 V produces nearly rectangular CV curves at every scan rate, indicating good rate capacity and electrochemical reversibility of the device. Figure 8(c) shows the GCD curves at various current densities, and the calculated areal capacitances of this symmetric supercapacitor according to eq 2 at various current densities are illustrated in Figure 8(d). As shown in Figure 8(c), the GCD curves demonstrate nearly symmetric and linear shapes at various current densities, indicating the capacitive behavior of this screen-printed supercapacitor on a Spandex fabric. For the as-prepared supercapacitor, the highest areal capacitance of 33.8 mF cm^–2^ was obtained at a 0.2 mA cm^–2^ current density, and the device maintains a capacitance of 14.1 mF cm^–2^ even at a higher current density of 2.0 mA cm^–2^, indicating the superior rate capability of the device. The relationship between areal capacitance and current densities (Figure 8(d) indicates that the areal capacitance of the supercapacitor drops with the increase in current density because electrolyte ions cannot flow through electrode pores easily at high current densities due to the insufficient reaction time.^30,64^ The Nyquist plot shown in Figure 8(e) shows a fairly straight line in the low-frequency region, which reflects the diffusion-controlled Warburg impedance at the electrode/electrolyte interface, and a small semicircle in the high-frequency region, which corresponds to charge-transfer resistance (*R*_ct_). An equivalent circuit model was employed to fit the impedance curve shown in Figure 8(f), where *R*_s_ stands for equivalent series resistance, *C*_dl_ for the double-layer capacitor, *R*_ct_ for interfacial charge-transfer resistance, *W* for Warburg impedance, and CPE for constant phase element (faradic pseudocapacitance). *R*_s_ and *R*_ct_ values are extracted after the circuit fitting, which were found to be ∼2.98 and 1.06 Ω, respectively. The lower value values of the *R*_s_ and *R*_ct_ indicate excellent electrical contact and fast ionic transport at the electrode/electrolyte interface. In the low-frequency zone, a nearly vertical line is observed, which signifies the capacitive behavior of the device.

**Figure 9:**
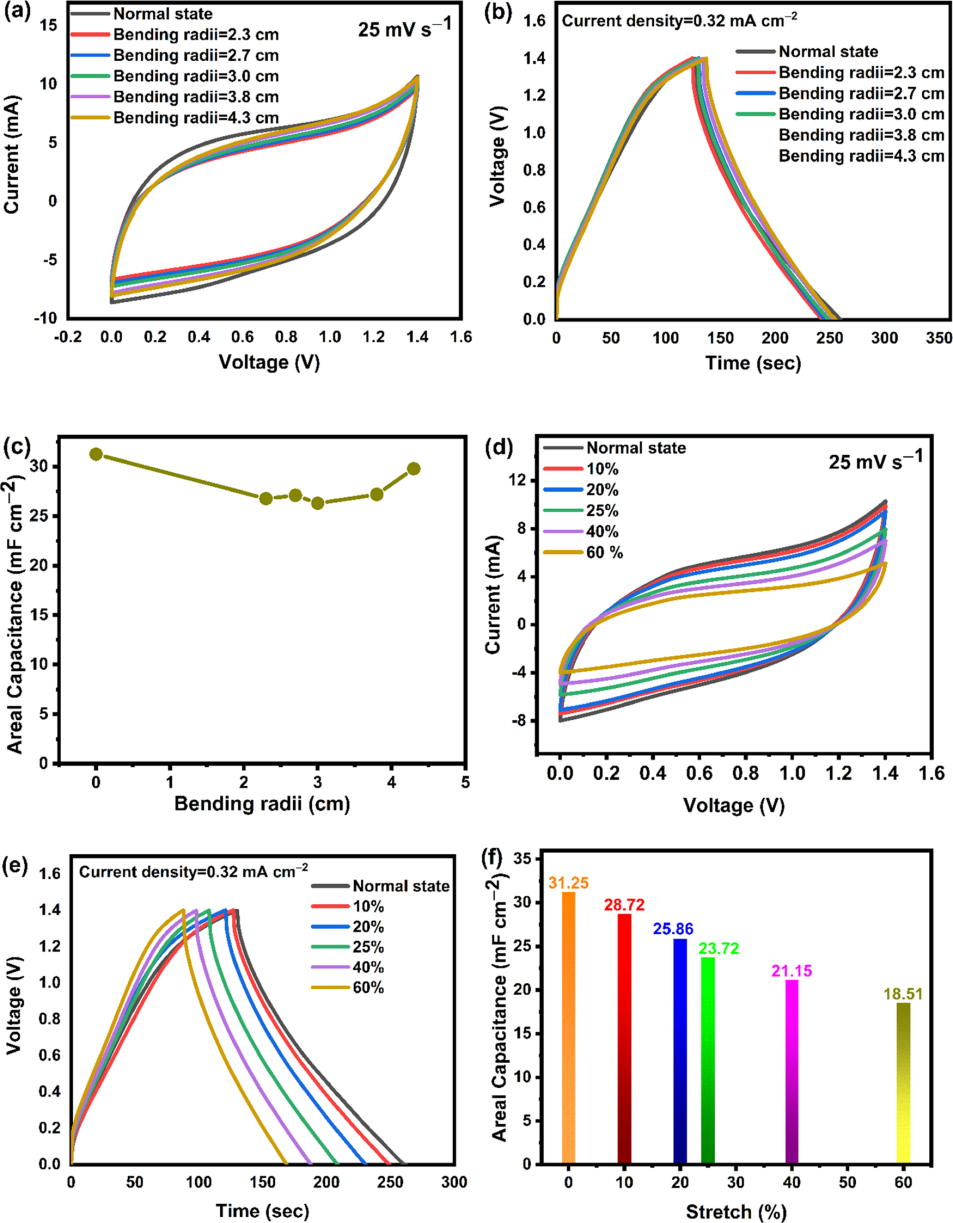
(a) CV at a 25 mV s^–1^ scan rate. (b) GCD at a 0.32 mA cm^–2^ current density. (c) Areal capacitance vs bending radii of the screen-printed ACFM-850/TS-based interdigitated symmetric supercapacitor under various bending conditions. (d) CV curves at a 25 mV s^–1^ scan rate under different stretching conditions (0–60%). (e) GCD curves at a 0.32 mA cm^–2^ current density. (f) Areal capacitance vs % stretch of the screen-printed ACFM-850/TS-based interdigitated symmetric supercapacitor under different stretching conditions.

To further demonstrate the stability and mechanical strength of this supercapacitor for practical applications, electrochemical tests of the ACFM-850/TS-based symmetric supercapacitor fabricated on a stretchable fabric were evaluated under different bending and stretching conditions, as shown in Figure 9. Figure S8a (Supporting Information) shows the bending tests carried out by attaching the fabricated device to glass beakers of different diameters. For the as-prepared device, the CV and GCD curves shown in Figure 9(a,b) are nearly identical under different bending conditions. Additionally, as shown in Figure 9(c), there is only a very slight capacitance loss (20%) under various bending situations, demonstrating the high mechanical stability of this device. Figure S8b shows the photographs of the device fabricated on the Spandex fabric stretched to different lengths. The electrochemical behavior of the screen-printed supercapacitor was tested under different stretching conditions from 0 to 60%, as shown in Figure 9(d–f). As shown in Figure 9(d), the CV curves are practically the same until 20% stretching, but the current response started to reduce from 25 to 60% stretching. However, even under extreme stretching conditions, the device maintained a quasi-rectangular shape, demonstrating the remarkable electrochemical properties of this stretchable supercapacitor. Figure 9(e,f), respectively, shows the GCD curves and related areal specific capacitances. The GCD curves at different stretching scenarios displayed nearly linear charge/discharge behavior, retaining 59% of its initial capacitance under 60% stretching. The excellent stretchable stability of this device further highlights its potential for real-time applications of the screen-printed stretchable supercapacitor fabricated with ACFM-850/TS composite ink as electroactive material using CMC/NaClO_4_ as an electrolyte.

**Figure 10:**
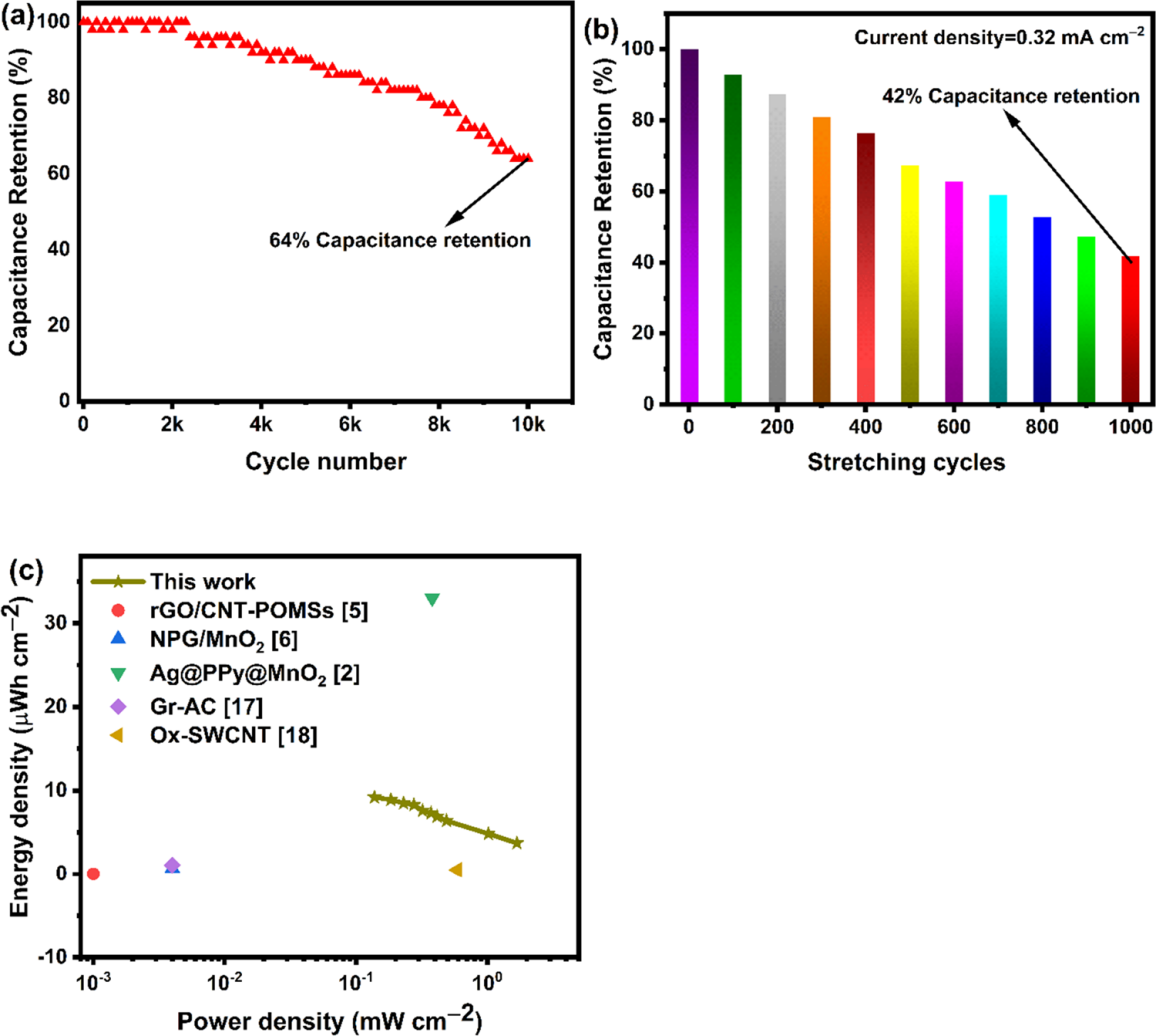
(a) Percentage of capacitance retained as a function of the number of charge–discharge cycles under a normal state of operation at a current density of 0.66 mA cm^–2^. (b) Percentage of capacitance retained as a function of the cycle number under 25% stretching at a 0.32 mA cm^–2^ current density. (c) Areal energy and power density (Ragone) plot of this work compared with previously published interdigitated supercapacitors.

Figure 10(a) shows the cycling performance of the as-prepared ACFM-850/TS-based interdigitated symmetric supercapacitor at a high current density of 0.66 mA cm^–2^. The interdigitated supercapacitor retains 64% of its initial capacitance after 10,000 charge–discharge cycles, demonstrating that this supercapacitor delivers long service life and ultrastable capacity. The cyclic stability of the as-prepared ACFM-850/TS-based interdigitated symmetric supercapacitor reported in this work is greater than the activated carbon on flexible nickel foam-based symmetric supercapacitor fabricated by Tu et al.,^37^ which retained 51.4% after 5000 cycles. Furthermore, we investigated the capacitance retention performance of the supercapacitor during multiple stretch/release cycles, as shown in Figure S9. GCD curves are recorded for every 100 stretch/release cycles and the performance has been calculated. It can be observed from Figure 10(b) that the device maintained 42% of its capacitance even after 1000 stretch/release cycles at 25% stretching relative to the capacitance of the supercapacitor after the first stretch/release cycle. Figure 10(c) shows the Ragone plot of the areal energy density versus power density. The screen-printed ACFM-850/TS composite-based interdigitated supercapacitor exhibited the maximum areal energy and power densities of 9.2 μWh cm^–2^ and 1.67 mW cm^–2^, respectively. Compared with other reported screen-printed and 3D-printed interdigitated supercapacitors, the screen-printed ACFM-850/TS composite-based interdigitated supercapacitor exhibited good areal energy density and power density performances, which are comparable/better than interdigitated supercapacitors reported in the literature.^2,5,6,17,18^ These results impart significant application prospects to the as-fabricated ACFM-850/TS composite-based interdigitated supercapacitor with excellent performances.

## 4 Conclusions

In summary, we have demonstrated the application of a face-mask-derived activated carbon/tin sulfide composite material as a high-performance electrode material for supercapacitor application. Tin sulfide (TS) sheets are directly anchored inside and on the high-surface-area porous carbon (ACFM-850) using a straightforward hydrothermal process, significantly improving the composite’s electron transport channels and increasing the energy-storage capability of the composite. Due to the combined effect of ACFM-850 and TS materials, the specific capacitance of the ACFM-850/TS composite reached up to 423 F g^–1^ at a 0.5 A g^–1^ current density, with an excellent rate capability of 71.3% capacitance retention at a high current density 30 A g^–1^ in a 1.0 M Na_2_SO_4_ electrolyte. Furthermore, we fabricated an interdigitated symmetric supercapacitor on a stretchable Spandex fabric using an ACFM-850/TS composite electrode and CMC/NaClO_4_ as an electrolyte. The fully screen-printed interdigitated supercapacitor has an exceptional energy density of 9.2 μWh cm^–2^ at a power density of 0.13 mW cm^–2^ and a high retention ratio of 64% of its initial capacitance after 10,000 cycles. This face-mask-derived activated carbon/tin sulfide composite (ACFM-850/TS) with excellent electrochemical performance reported in this work acts as a promising electrode material for fabricating screen-printed supercapacitors, which could be used in next-generation energy-storage and wearable electronics applications. Furthermore, this work provides a low-cost, cost-effective, and sustainable synthetic strategy for converting used polypropylene face masks into valuable carbon-based products for real-time supercapacitor applications.

## Supporting Information Available

Raman spectra and deconvoluted Raman spectra of ACFM-T samples prepared at different activation temperatures (Figure S1), high-resolution TEM images of the ACFM-850 sample at different magnifications (Figure S2), XPS survey spectra of ACFM-T samples and high-resolution deconvoluted XPS spectra of ACFM-T samples prepared at different activation temperatures (Figure S3), electrochemical performance of ACFM-T electrodes measured in a 1 M Na_2_SO_4_ electrolyte in a three-electrode system (Figure S4), electrochemical performance of the tin sulfide (TS) electrode measured in a 1 M Na_2_SO_4_ electrolyte in a three-electrode system (Figure S5), electrochemical performance of three different ACFM-850/TS electrodes tested in a 1 M Na_2_SO_4_ aqueous electrolyte (Figure S6), GCD curve of the symmetric supercapacitor device measured at a 0.20 mA cm^–2^ current density (Figure S7), photographs of the ACFM-850/TS composite-based interdigitated supercapacitor subjected to different bending and stretching conditions (Figure S8), photographs of the ACFM-850/TS composite-based interdigitated supercapacitor after 1st and 1000th cycle at 25% stretching along with its CV behavior (Figure S9), and performance of three different ACFM-850/TS electrodes a 1 M Na_2_SO_4_ aqueous electrolyte (Table S1) (PDF)
