# Supplementary material for: Screen-Printed Stretchable Supercapacitors Based on Tin Sulfide-Decorated Face-Mask-Derived Activated Carbon Electrodes with High Areal Energy Density
Source: ACS Appl Energy Mater. 2024 Apr 18;7(9):3558–76. doi: 10.1021/acsaem.3c02902 (PMC11094728; doi:10.1021/acsaem.3c02902)
Supplement: Supplementary file 1 — ae3c02902_si_001.pdf [file ae3c02902_si_001.pdf]

Supporting Information

Screen printed stretchable supercapacitors based on tin sulfide decorated facemask derived activated carbon electrodes with high areal energy density

Kiran Kumar Reddy Reddygunta,[a] *Lidija Šiller,*[b] *and Aruna Ivaturi*[a*]

[a]Smart Materials Research and Device Technology (SMaRDT) Group, Department of Pure and Applied Chemistry, University of Strathclyde, Thomas Graham Building, Glasgow, G1 1XL, UK

[b]School of Engineering, Newcastle University, Newcastle upon Tyne, NE1 7RU, UK

Corresponding author email: aruna.ivaturi@strath.ac.uk

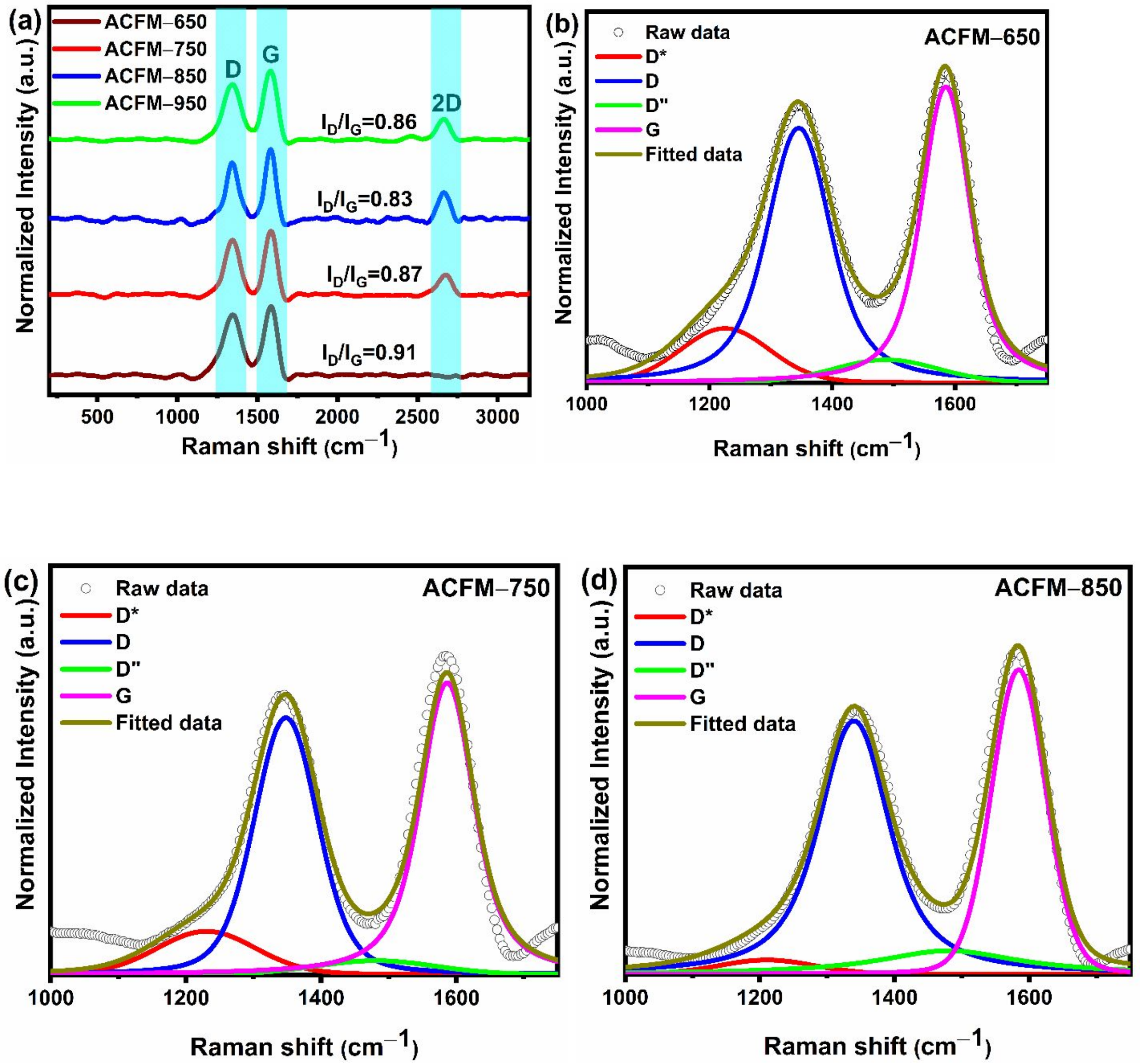
(a)
ACFM−650
ACFM−750
ACFM−850
ACFM−950
D
G
2D
I_D/I_G=0.86
I_D/I_G=0.83
I_D/I_G=0.87
I_D/I_G=0.91
Normalized Intensity (a.u.)
Raman shift (cm^{-1})
(b)
Raw data
D*
D
D''
G
Fitted data
ACFM−650
(c)
ACFM−750
(d)
ACFM−850

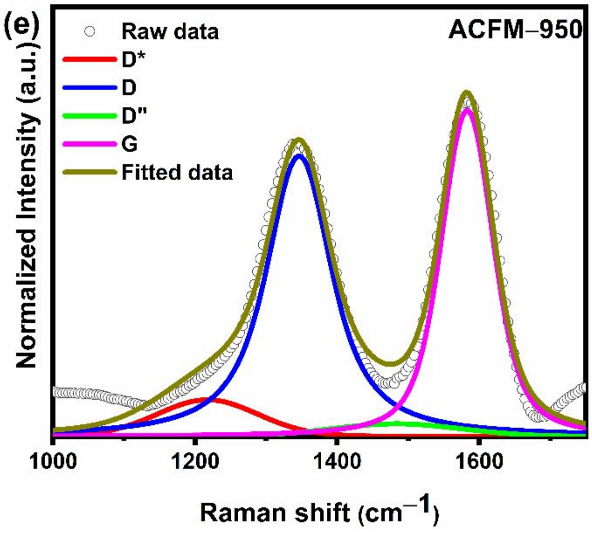

Figure S1. (a) Raman spectra of ACFM–T samples prepared at different activation temperatures; Deconvoluted Raman spectra of (b) ACFM–650 (c) ACFM–750 (d) ACFM–850 and (e) ACFM–950 samples

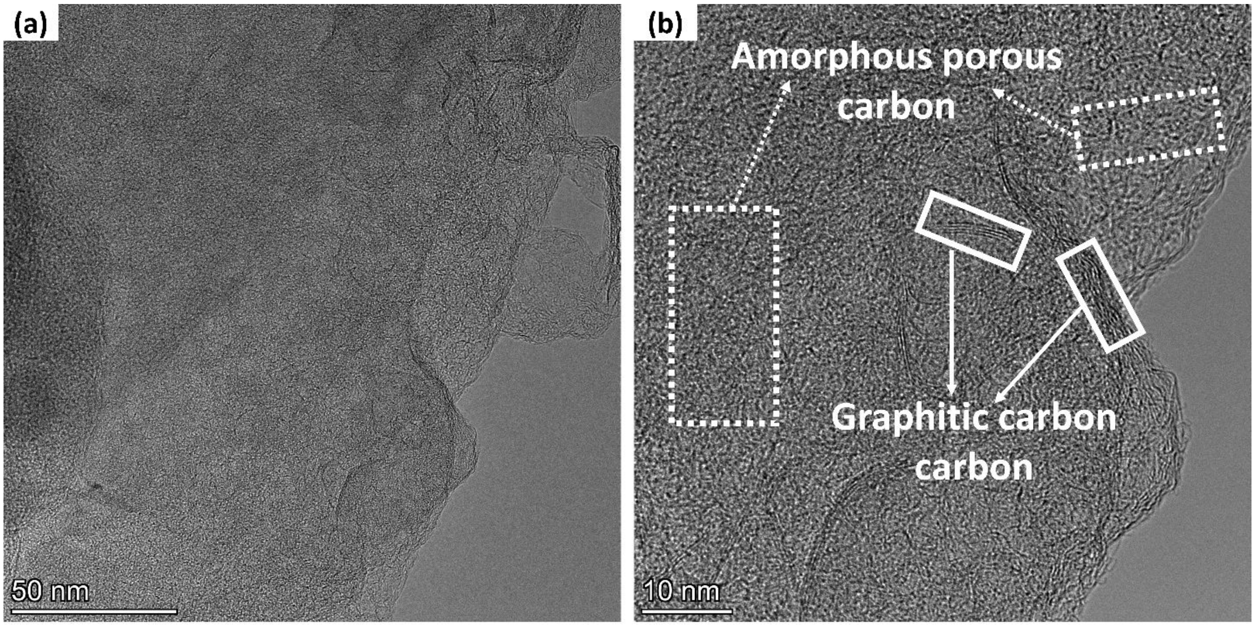

Figure S2. (a, b) High-Resolution TEM images of ACFM–850 sample at different magnifications

The overall XPS survey scan of face mask derived based activated carbons (ACFM–T) mainly comprises of C 1s, N 1s and O 1s peaks at 284.5 eV, 400 eV and 532.5 eV with trace amount of sulfur (S 2p) peak located at ~165 eV, respectively. The XPS studies show that carbon is the predominant element in all the corn based activated carbons with small amounts of oxygen, nitrogen, and sulfur also present, most probably on the surface of the carbon framework.

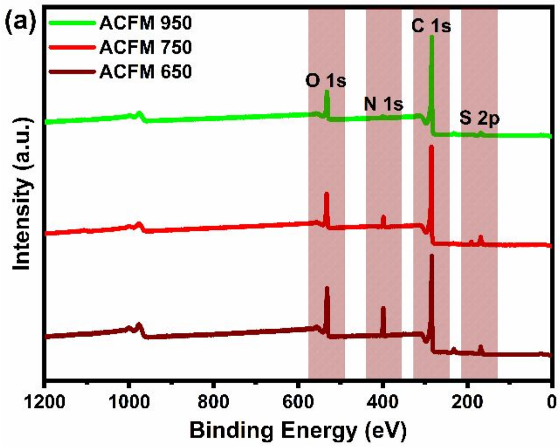

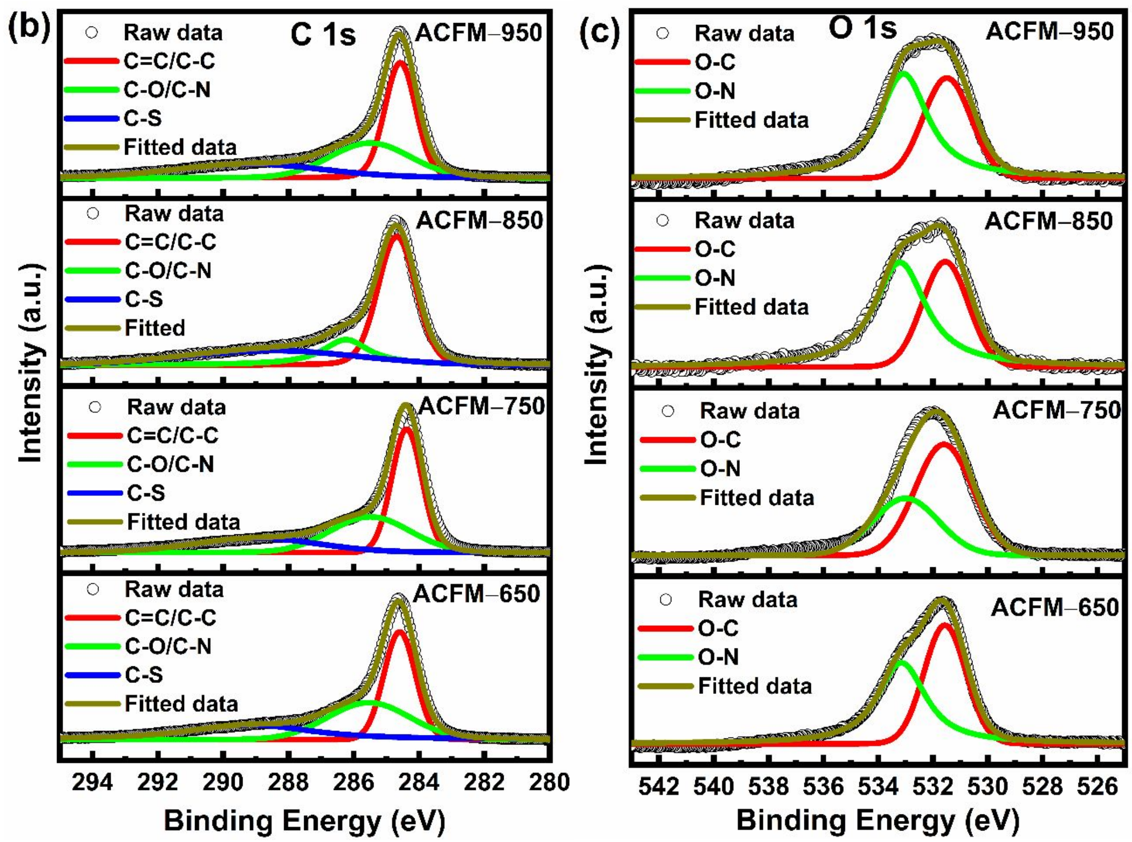
(b)
C 1s
Raw data
C=C/C-C
C-O/C-N
C-S
Fitted data
ACFM−950
Raw data
C=C/C-C
C-O/C-N
C-S
Fitted
ACFM−850
Raw data
C=C/C-C
C-O/C-N
C-S
Fitted data
ACFM−750
Raw data
C=C/C-C
C-O/C-N
C-S
Fitted data
ACFM−650
Intensity (a.u.)
294 292 290 288 286 284 282 280
Binding Energy (eV)
(c)
O 1s
Raw data
O-C
O-N
Fitted data
ACFM−950
ACFM−850
ACFM−750
ACFM−650
542 540 538 536 534 532 530 528 526

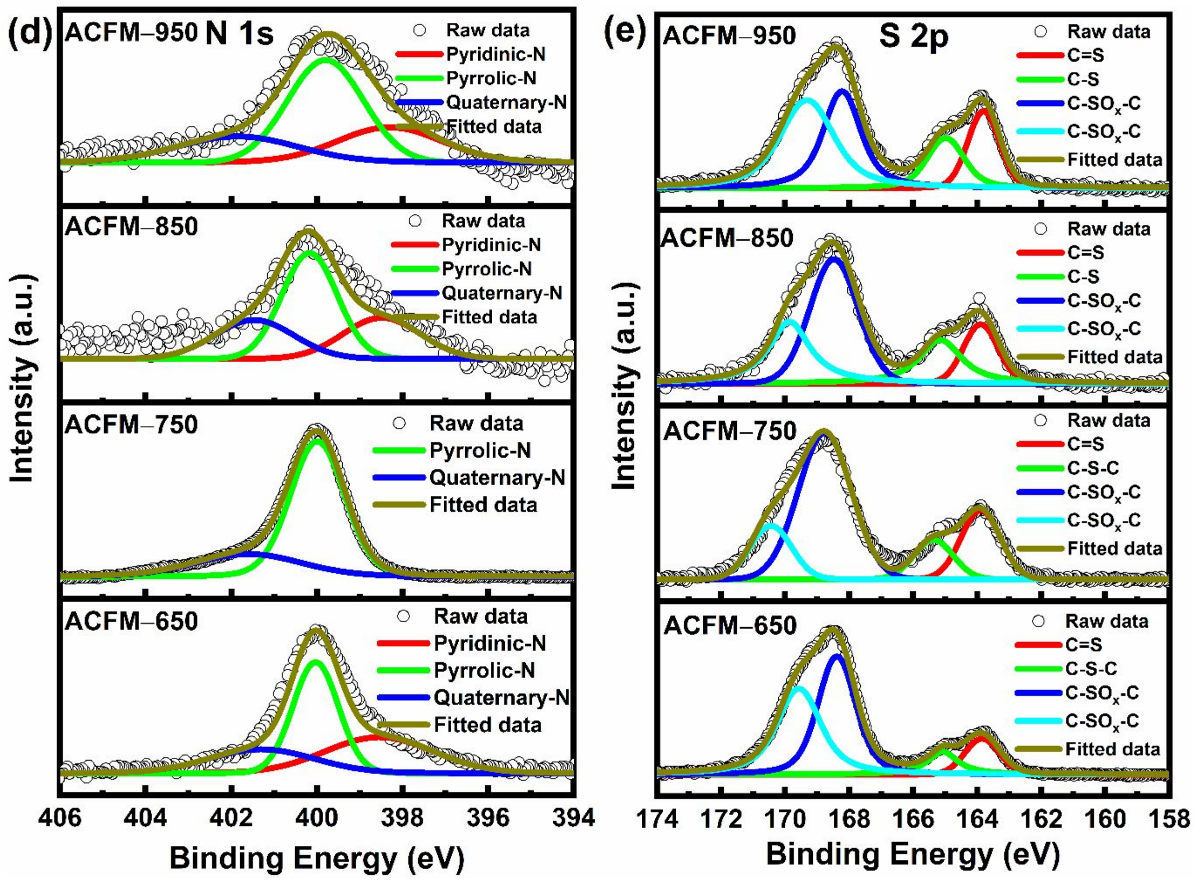

Figure S3. (a) XPS survey spectra of ACFM–T samples prepared at different activation temperatures. High resolution deconvoluted XPS spectra of (b) C 1s (c) O 1s (d) N 1s and (e) S 2p spectra of ACFM–T samples prepared at different activation temperatures.

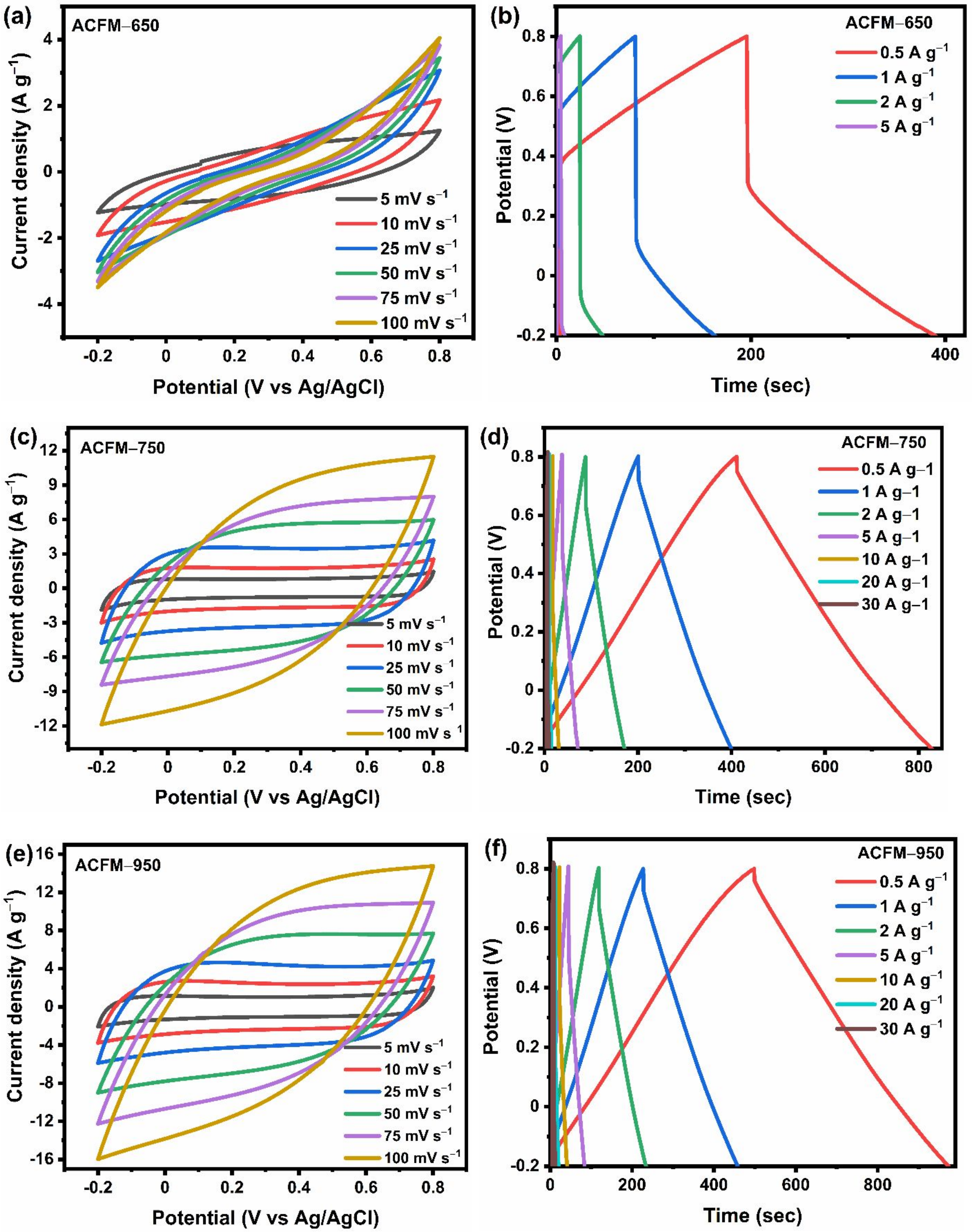

(a)
ACFM–650
Current density (A g^{-1})
Potential (V vs Ag/AgCl)
5 mV s^{-1}
10 mV s^{-1}
25 mV s^{-1}
50 mV s^{-1}
75 mV s^{-1}
100 mV s^{-1}
(b)
ACFM–650
Potential (V)
Time (sec)
0.5 A g^{-1}
1 A g^{-1}
2 A g^{-1}
5 A g^{-1}
(c)
ACFM–750
Current density (A g^{-1})
Potential (V vs Ag/AgCl)
5 mV s^{-1}
10 mV s^{-1}
25 mV s^{-1}
50 mV s^{-1}
75 mV s^{-1}
100 mV s^{-1}
(d)
ACFM–750
Potential (V)
Time (sec)
0.5 A g–1
1 A g–1
2 A g–1
5 A g–1
10 A g–1
20 A g–1
30 A g–1
(e)
ACFM–950
Current density (A g^{-1})
Potential (V vs Ag/AgCl)
5 mV s^{-1}
10 mV s^{-1}
25 mV s^{-1}
50 mV s^{-1}
75 mV s^{-1}
100 mV s^{-1}
(f)
ACFM–950
Potential (V)
Time (sec)
0.5 A g^{-1}
1 A g^{-1}
2 A g^{-1}
5 A g^{-1}
10 A g^{-1}
20 A g^{-1}
30 A g^{-1}

Figure S4. (a) CV curves of ACFM−650 electrode at different scan rates (b) GCD curves of ACFM−650 electrode at different current densities (c) CV curves of ACFM−750 electrode at different scan rates (d) GCD curves of ACFM−750 electrode at different current densities (e) CV curves of ACFM−950 electrode at different scan rates (f) GCD curves of ACFM−950 electrode at different current densities measured in 1 M Na_2SO_4 electrolyte in three electrode system

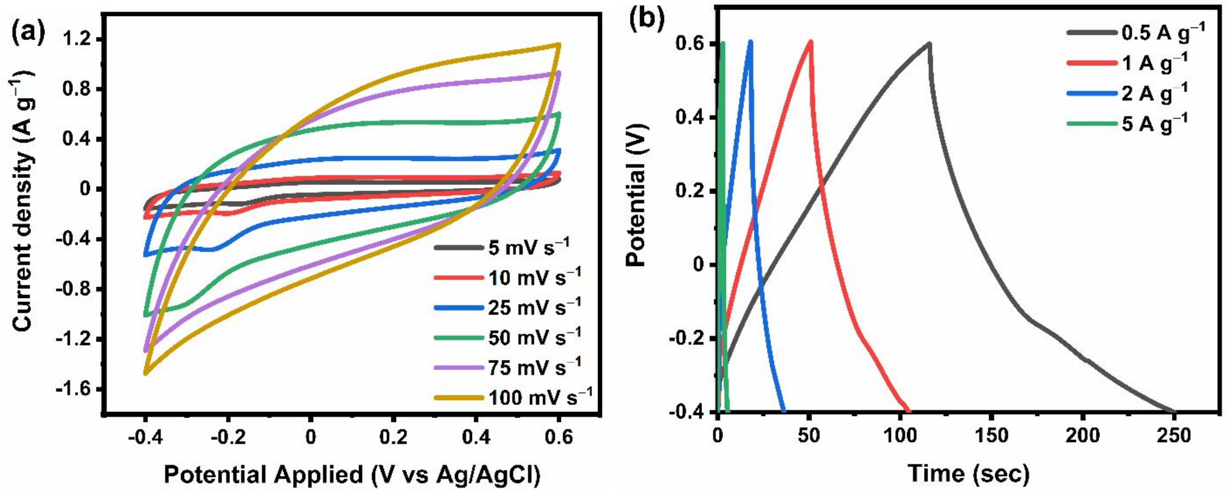

Figure S5. (a) CV curves at different scan rates (b) GCD curves of at different current densities, of Tin sulfide (T.S) electrode measured in 1 M Na_2SO_4 electrolyte in three electrode system.

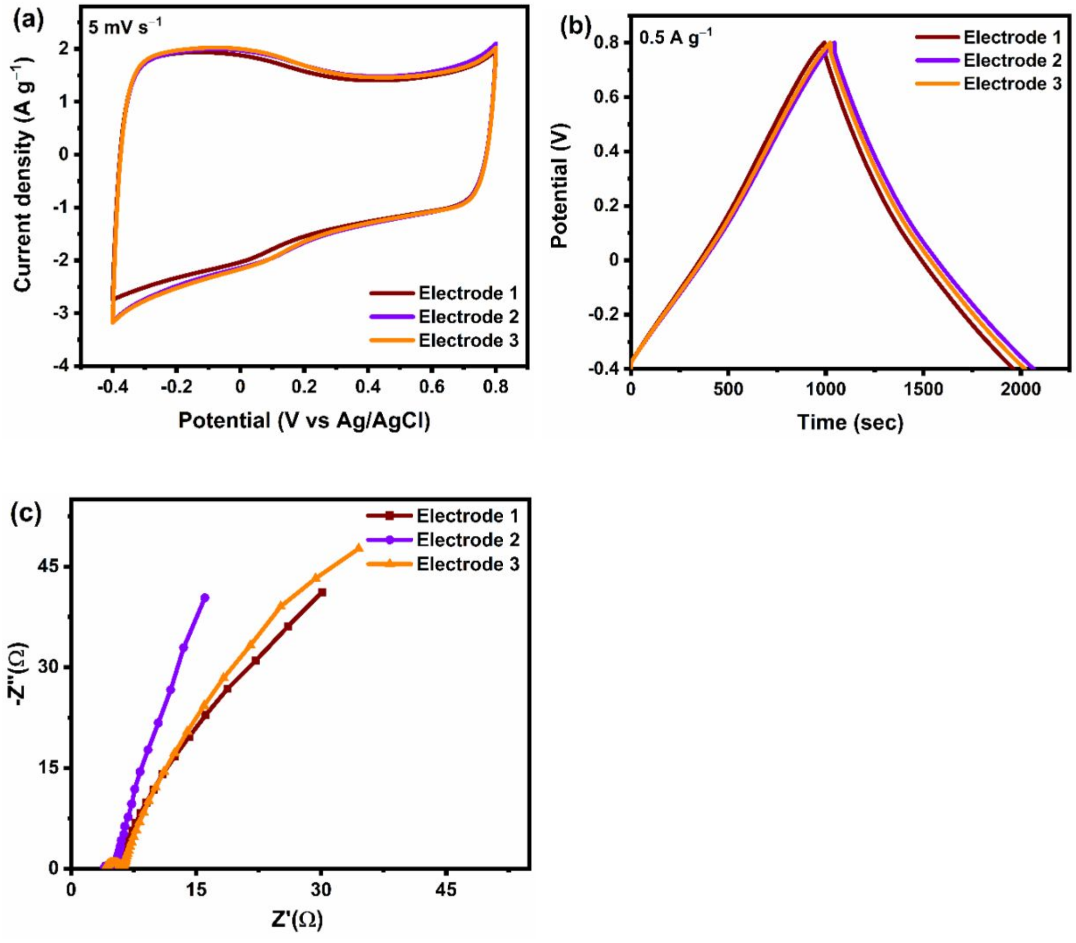

Figure S6. (a) CV curves (b) GCD curves (c) Nyquist plots of three different ACFM–850/T.S electrodes tested in 1 M Na_2SO_4 aqueous electrolyte

Table S1. Performance of three different ACFM–850/T.S electrodes 1 M Na_2SO_4 aqueous electrolyte

**Parameters**	**Electrode 1**	**Electrode 2**	**Electrode 3**
Sample weight	2.1 mg	2.3 mg	2.6 mg
Specific capacitance (F g^{-1}) at 0.5 A g^{-1} current density	402	423	414
R_s (Ω)	3.9	3.9	4.7
R_{ct} (Ω)	1.2	1.7	1.9

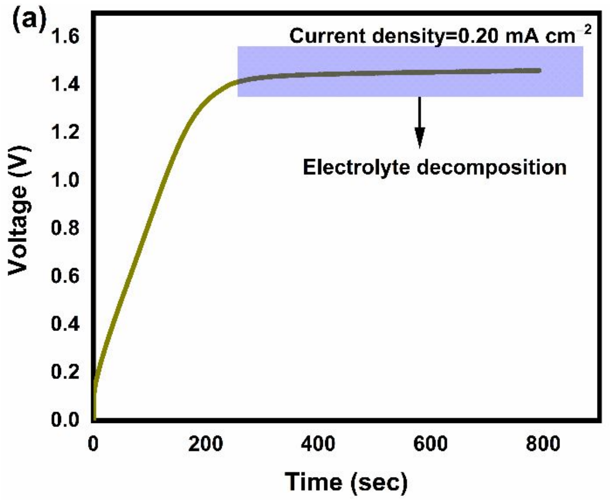

Figure S7. (a) GCD curve of symmetric supercapacitor device measured at 0.20 mA cm^{-2} current density

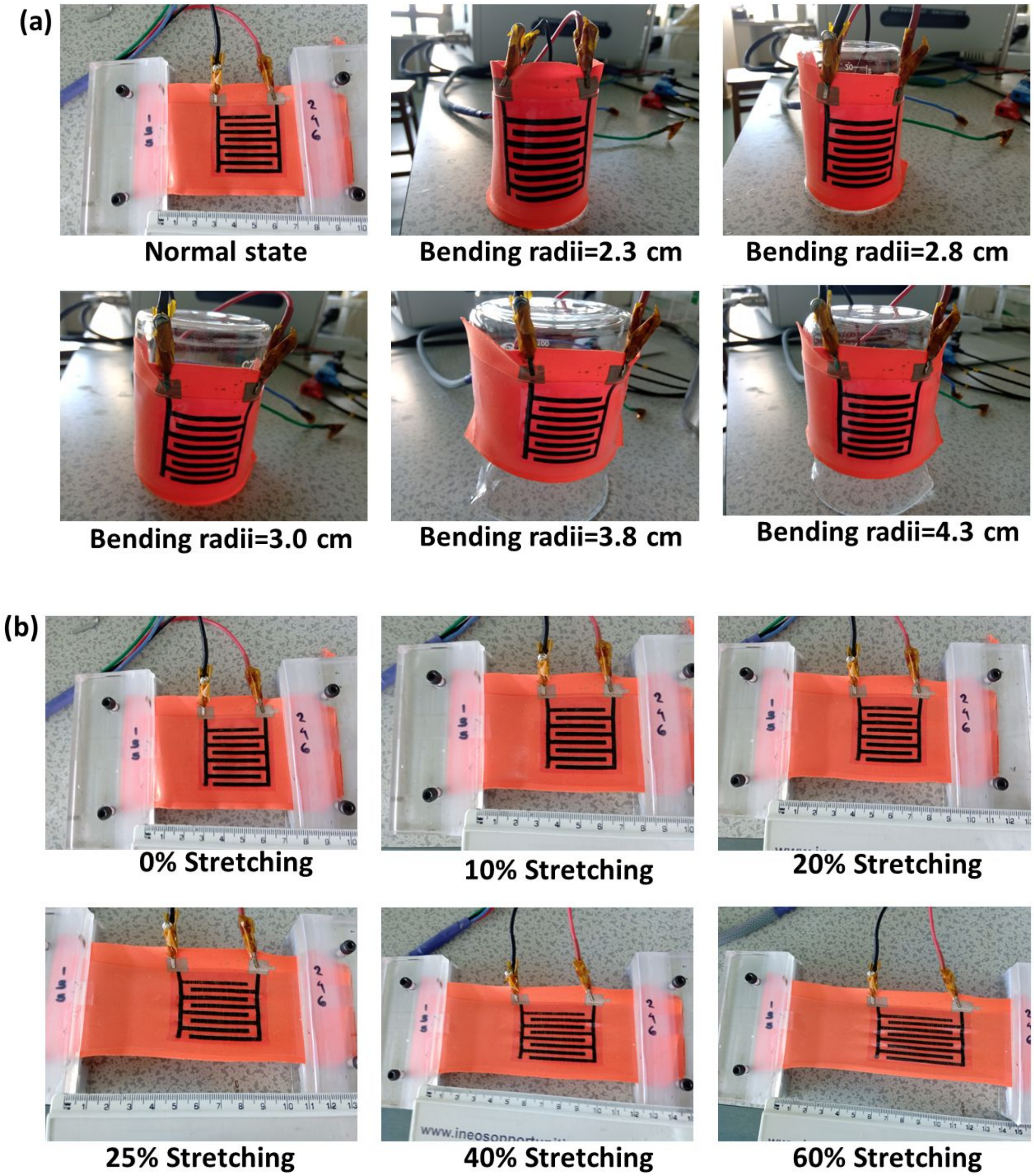

Figure S8. Photographs of ACFM-850/T.S composite based interdigitated supercapacitor subjected to different (a) bending and (b) stretching conditions

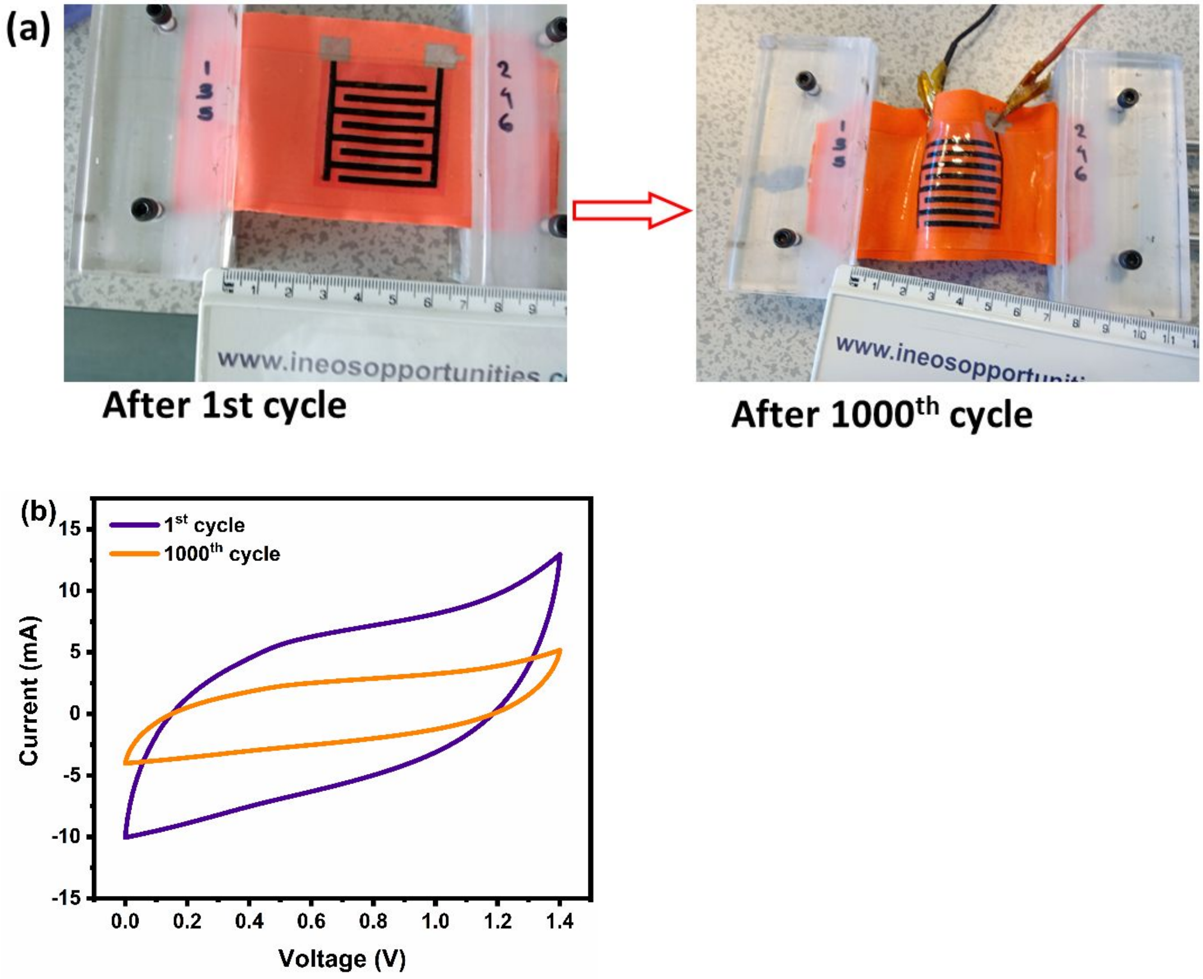

Figure S9. (a) Photographs of ACFM-850/T.S composite based interdigitated supercapacitor after 1st and 1000th cycle at 25% stretching (b) CV curves of ACFM-850/T.S composite based interdigitated supercapacitor after 1st and 1000th cycle at 25% stretching at 5 mV s^{-1} scan rate
